# Prdx1 Interacts with ASK1 upon Exposure to H_2_O_2_ and Independently of a Scaffolding Protein

**DOI:** 10.3390/antiox10071060

**Published:** 2021-06-30

**Authors:** Trung Nghia Vo, Julia Malo Pueyo, Khadija Wahni, Daria Ezeriņa, Jesalyn Bolduc, Joris Messens

**Affiliations:** 1 VIB-VUB Center for Structural Biology, Vlaams Instituut Voor Biotechnologie, B-1050 Brussels, Belgium; trung.nghia.vo@vub.be (T.N.V.); julia.malo.pueyo@vub.be (J.M.P.); khadija.wahni@vub.be (K.W.); daria.ezerina@vub.be (D.E.); jesalyn.bolduc@vub.be (J.B.); 2 Brussels Center for Redox Biology, Vrije Universiteit Brussel, B-1050 Brussels, Belgium; 3 Structural Biology Brussels, Vrije Universiteit Brussel, B-1050 Brussels, Belgium

**Keywords:** peroxiredoxin, redox communication, hydrogen peroxide, Prx1, Prdx1, Prdx2, Prx2, annexin A2, ASK1

## Abstract

Hydrogen peroxide (H_2_O_2_) is a key redox signaling molecule that selectively oxidizes cysteines on proteins. It can accomplish this even in the presence of highly efficient and abundant H_2_O_2_ scavengers, peroxiredoxins (Prdxs), as it is the Prdxs themselves that transfer oxidative equivalents to specific protein thiols on target proteins via their redox-relay functionality. The first evidence of a mammalian cytosolic Prdx-mediated redox-relay—Prdx1 with the kinase ASK1—was presented a decade ago based on the outcome of a co-immunoprecipitation experiment. A second such redox-relay—Prdx2:STAT3—soon followed, for which further studies provided insights into its specificity, organization, and mechanism. The Prdx1:ASK1 redox-relay, however, has never undergone such a characterization. Here, we combine cellular and in vitro protein–protein interaction methods to investigate the Prdx1:ASK1 interaction more thoroughly. We show that, contrary to the Prdx2:STAT3 redox-relay, Prdx1 interacts with ASK1 at elevated H_2_O_2_ concentrations, and that this interaction can happen independently of a scaffolding protein. We also provide evidence of a Prdx2:ASK1 interaction, and demonstrate that it requires a facilitator that, however, is not annexin A2. Our results reveal that cytosolic Prdx redox-relays can be organized in different ways and yet again highlight the differentiated roles of Prdx1 and Prdx2.

## 1. Introduction

Hydrogen peroxide (H_2_O_2_) is a major reactive oxygen species (ROS) that acts as an intracellular signaling molecule by selective oxidation of cysteines on proteins. In doing so, H_2_O_2_ regulates several biological activities, such as cell proliferation and differentiation, tissue repair, inflammation, the circadian rhythm, and aging [1]. The major endogenous H_2_O_2_ sources are the mitochondrial respiratory chain and the NADPH oxidases (NOX), which release H_2_O_2_ in a regulated, localized manner [2,3,4]. To ensure that H_2_O_2_ levels stay within the nanomolar range and it does not accumulate to levels where it may exert irreversible damage to biomolecules, cells also harbor H_2_O_2_ scavenging systems. These include catalases, glutathione peroxidases (Gpxs), and peroxiredoxins (Prdxs) [1,3,5]. Among them, Prdxs are responsible for the majority of H_2_O_2_ reduction within cells as Prdxs are highly abundant (≈1% of total soluble protein content) and very efficient in removing peroxides (k ≈ 10^5^–10^8^ M^−1^ s^−1^) [3,5,6,7]. Prdxs scavenge H_2_O_2_ through the formation of a sulfenic acid (Cys-SOH) on the peroxidatic cysteine (Cys_P_) with the subsequent formation of a disulfide bond between Cys_P_ and the resolving cysteine (Cys_R_) [8], and are recycled via the thioredoxin pathway (Trx-TrxR-NADPH) [9]. This super-scavenging activity is due to the low pKa of the Cys_P_ and a conserved hydrogen bonding network which renders one of the oxygens of H_2_O_2_ more susceptible to nucleophilic attack by Cys_P_ [10]. In the presence of such abundant and highly efficient H_2_O_2_ scavengers, for a long time it was unclear how even some low-abundant proteins, whose thiols typically have kinetic rate constants in the 10–10^2^ M^−1^ s^−1^ range [5], can get oxidized [11,12]. Accumulating evidence [13,14,15,16] suggests that the answer to this conundrum lies in the ability of the sulfenic acid formed on Cys_P_ of Prdxs to react with a thiol of a target protein, instead of Cys_R_, or of the Cys_P_–Cys_R_ disulfide to partake in thiol–disulfide exchange with nucleophilic thiols of target proteins. Both lead to the formation of a mixed disulfide with the target protein via which the oxidative equivalents are transferred in a process termed “redox-relay”. This mixed disulfide can then rearrange into an intramolecular disulfide on the target protein, which can affect the protein’s localization, conformation, interaction partners, or functionality [17,18,19,20,21,22,23].

**Figure 1:**
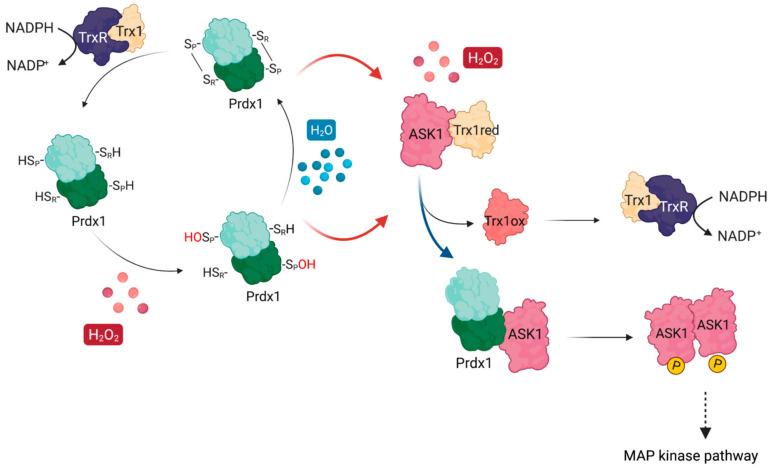
ASK1 is redox-regulated by Prdx1 in the presence of H_2_O_2_. At basal H_2_O_2_ levels, reduced Trx1 binds ASK1 non-covalently at its TBD, shielding reactive cysteines (e.g., Cys250). At increasing H_2_O_2_ levels, H_2_O_2_ oxidizes Prdx1 and leads to the release of oxidized Trx1 from ASK1, exposing the reactive cysteines. Oxidized Prdx1 then transfers oxidative equivalents to ASK1 (red arrows), wherein ASK1 performs a nucleophilic attack either on the intramolecular disulfide bond of Prdx2, or on the sulfenylated Cys_P_, leading to the formation of ASK1 disulfide-linked multimers, which are required for its kinase activity. Activated ASK1 becomes autophosphorylated, and in turn phosphorylates MAPKK, activating the p38/JNK signaling pathway, which leads to the expression of regulatory genes related with apoptosis, cell cycle regulation, and inflammation. Oxidized Trx1 is reduced via TrxR/NADPH.

The first documented example of such redox-relays came from budding yeast, where the thiol peroxidase Orp1 was shown to specifically transfer oxidative equivalents to the transcription factor Yap1 through a direct protein–protein interaction (PPI) in the presence of H_2_O_2_. As a result of this oxidation by Orp1, Yap1 is retained in the nucleus launching an adaptive transcriptional program [13]. Ten years after the discovery of the Orp1:Yap1 redox-relay, the first evidence of such redox-relays in the cytosol of mammalian cells was reported by Jarvis et al. [14], who demonstrated that Prdx1 facilitates the oxidation of the apoptosis signal-regulating kinase 1 (ASK1). ASK1 is a mitogen-activated protein kinase kinase kinase (MAP3K) which phosphorylates downstream kinases, eventually leading to the activation of the c-Jun N-terminal kinase (JNK) and p38 MAP kinase pathways [24,25,26]. Activation of these pathways, in turn, can result in the upregulation of important targets such as AP-1 (c-Jun family member), NFκB, and STAT1, all of which are implicated in cancer [27,28,29] (Figure 1). To exert its kinase activity, ASK1 needs to be oxidized to disulfide-bonded multimers, with Cys250 playing a critical role [30,31]. ASK1 multimers are then reduced by Trx1 [30]. In addition, reduced Trx1 regulates ASK1 activity by non-covalently binding to the so-called Trx-binding domain (ASK1-TBD) in the vicinity of Cys250 [25,32]. The inhibitory effect of this binding can be explained by the fact that it prevents ASK1 multimerization or the formation of other potential disulfide bonds that could alter its tertiary or quaternary structure and lead to activation with basal levels of H_2_O_2_ [31]. Upon exposure to an H_2_O_2_ signal (which can differ from basal levels by up to 50-fold, reaching 500–700 nM [33]), Trx1 becomes oxidized and dissociates from ASK1 (Figure 1), thereby allowing ASK1 to form disulfide-bonded multimers, become auto-phosphorylated, and unleash the ASK1-p38/JNK cascade [24,25,26]. The discovery of the Prdx1:ASK1 redox-relay, therefore, fit well with this model of H_2_O_2_-mediated ASK1 activation, with oxidized Prdx1 forming a putative mixed disulfide with Cys250 of ASK1 after the dissociation of Trx1 from ASK1-TBD.

Though the paper by Jarvis et al. (2012) [14] has been widely cited as the first Prdx-mediated redox-relay in the cytosol of mammalian cells, evidence for this relay is based on only a few experiments. The first is indirect: an attenuation of ASK1 oxidation and p38 phosphorylation was seen upon Prdx1 knock down. The second piece of evidence comes from the comparison of reducing and non-reducing immunoblots against ASK1, where mixed disulfides corresponding in size to ASK1:Prdx1 intermediates could be observed. A co-immunoprecipitation experiment on cell lysates then revealed that ASK1 co-precipitates with Prdx1, and that this mixed disulfide can be reduced by DTT [14]. Even though the authors took care in preventing artificial oxidation upon cell lysis by carrying out the experiment in the presence of high amounts (100 mM) of NEM, it was reported that thiol blocking under these conditions is not fully efficient [15,34]. To our knowledge, no further attempts of proving the Prdx1:ASK1 interaction by other means, including those that do not require cell lysis, have been undertaken.

In stark contrast, the second example of cytosolic Prdx-mediated redox-relays in mammalian cells, between Prdx2 and the transcription factor STAT3 (signal transducer and activator of transcription 3), received much more attention [15,35]. Indeed, the Prdx2:STAT3 redox has been confirmed in several experimental settings on cell lysates (co-immunoprecipitation), as well as in both fixed (proximity ligation assay) and live cells (bimolecular fluorescence complementation (BiFC) and Förster resonance energy transfer (FRET)). Together, these results gave more insight into the Prdx2:STAT3 interaction, revealing that Prdx2 and STAT3 already interact prior to and independent of H_2_O_2_, and are localized to membrane-associated microdomains, as well as this interaction being specific to Prdx2 over Prdx1. Interestingly, the only type of experiment where the Prdx2:STAT3 interaction was not observed was in vitro on recombinant proteins [35]. This discrepancy between in vitro and cellular experiments then led to the discovery that the transfer of oxidative equivalents from Prdx2 to STAT3 is facilitated by annexin A2 (AnxA2) [35], similar to how Ybp1 mediates the Orp1:Yap1 interaction in *S. cerevisiae* [36].

Inspired by the exciting insights into mammalian cytosolic Prdx redox-relays yielded by Prdx2:STAT3 interaction studies, in this study, we revisit the Prdx1:ASK1 redox-relay. We utilize BiFC as an approach for studying PPI in cells and bio-layer interferometry (BLI) as an in vitro PPI method to validate and gain more insight into the interaction reported a decade ago. Our results show that, unlike Prdx2 and STAT3, Prdx1 and ASK1 only interact upon elevation of cytosolic H_2_O_2_ levels above exogenous ones, and independently of a facilitator protein. Further, we found that Prdx2 can also bind to ASK1 in cells even after knocking out AnxA2, but not in vitro, suggesting the need for a facilitator protein that is not AnxA2.

## 2. Materials and Methods

### 2.1. Ab Initio Modeling by I-TASSER and Docking Simulations with HADDOCK

The I-TASSER (Iterative Threading Assembly Refinement) (Zhang Lab) webserver (https://zhanglab.ccmb.med.umich.edu/I-TASSER/, accessed in 30 November 2020) was used for the 3D ab initio modeling of the eight different Prdx1- and ASK1-mLumin fusion proteins [37]. The N- (LN) or C-terminal (LC) half of mLumin was fused to either the N- or C-terminus of Prdx1 and ASK1 separated by a linker. The linker for the LN fragment is: Gly-Ser-Tyr-Pro-Tyr-Asp-Val-Pro-Asp-Tyr-Ala-Gly-Thr-Gly-Gly-Ser-Lys-Ser-Thr; and for the LC is: Glu-Gln-Lys-Leu-Ile-Ser-Glu-Glu-Asp-Leu-Gly-Thr-Gly-Gly-Ser-Lys-Ser. The models obtained were superimposed with the human ASK1 kinase domain (PDB 2CLQ) [38] and the human Prdx1 C83S mutant (PDB 4XCS) [39] using PyMol (Version 2.4.1, Schrödinger, Inc., New York, USA) [40], and models where the secondary structure was maintained (low r.m.s.d.) were selected. These models were then submitted to the High Ambiguity Driven Biomolecular Docking (HADDOCK) (Bonvin Lab) [41] webserver (https://wenmr.science.uu.nl, accessed in 30 November 2020) to identify the most stable docking between Prdx1 and ASK1 fused to complementary mLumin fragments. The defined criteria for running the docking were: ‘All’ chains were defined to be involved in the interaction; the peroxidatic Cys52 of Prdx1 [42] and the Cys located in the Trx binding domain (TBD) (120, 185, 200, 206, 225, 226, and 250) of ASK1 [32] were specified as active residues; and residues surrounding the active residues were automatically defined as passive.

### 2.2. Cell Lines, Antibodies, Chemicals, and Plasticware

The HEK293 MSR (Griptite^TM^, Thermo Fisher Scientific. Waltham, WA, USA) and annexin A2 knock-out (AnxA2 KO) HEK293 MSR cells (gift from the lab of Tobias Dick, German Cancer Research Center, Heidelberg, Germany) [35] were maintained in Dulbecco’s Modified Eagle Medium (DMEM) (Life Technologies, Carlsbad, CA, USA), supplemented with 10% fetal bovine serum (FBS) (Life Technologies, Carlsbad, CA, USA) and 50 units/mL penicillin and streptomycin (P/S) (Life Technologies). Cell lines were routinely checked for the absence of mycoplasma using the PCR Mycoplasma Test Kit (PromoCell, Cat.PK-CA91-1024, Heidelberg, Germany). Plasticware for cell culture was from Avantor (Radnor, PA, USA).

Antibodies used in this study were rabbit anti-Prdx1 (D5G12) (8499, Cell Signaling Technology^®^, Danvers, MA, USA), mouse anti-Prdx2 (WH0007001M1, Sigma-Aldrich, St. Louis, MO, USA), mouse anti-ASK1 (MA5-15861, ThermoFisher Scientific), mouse anti-STAT3 (9139S, Cell Signaling Technology^®^, Danvers, Massachusetts, USA), mouse anti-His (AD1.1.10, Bio-Rad Laboratories, Richmond, VA, USA), rabbit anti-β-tubulin (2146S, Cell Signaling Technology^®^, Danvers, MA, USA), and mouse anti-β-actin (A2228, Sigma-Aldrich, St. Louis, MO, USA). All antibodies were used at a dilution of 1:1000 in Tris-buffered saline with 0.1% Tween 20 (TBS-T), except anti-Prdx2 which was used at a dilution of 1:5000 in TBS-T. The secondary antibodies for Western blot used in this study were anti-mouse IgG (A3562, Sigma-Aldrich, St. Louis, MO, USA) and anti-rabbit IgG (A8025, Sigma-Aldrich, St. Louis, MO, USA). All secondary antibodies were alkaline phosphatase conjugated and used at a dilution of 1:10000, also in TBS-T. The protein ladder used in SDS-PAGE was PageRuler Prestained protein ladder (Thermo Fisher Scientific, Waltham, WA, USA).

All chemicals were purchased from Merck (Sigma-Aldrich, St. Louis, MO, USA) unless stated otherwise.

### 2.3. Cloning of Prdx1, Prdx2, ASK1, and STAT3 DNA Constructs

The mammalian cell expression vector pcDNA3.1(−) harboring either the N- (LN) or C-terminal (LC) half of mLumin with the respective linker and the pcDNA3.1(−)-STAT3 LN, pcDNA3.1(−)-Prdx2 WT-LC, and pMAX-GFP constructs were kindly provided by the lab of Tobias Dick (German Cancer Research Center, Heidelberg, Germany). Prdx1 and ASK1 genes (codon-optimized for mammalian cells) synthesized at Genscript (Piscataway, NJ, USA) were cloned into the pcDNA3.1(−)-mLumin vectors using the NEBuilder^®^ HiFi DNA Assembly Master Mix (NEB Cat. E2621) (New England Biolabs, Ipswich, MA, USA), in the orientation predicted by the in silico modeling and docking simulations. Primers for the HiFi DNA Assembly were designed using Snapgene (version 5.2.2. GSL Biotech. San Diego, CA, USA). Peroxidatic Cys (C52A) and resolving Cys (C173A) mutants of Prdx1 were generated by site-directed mutagenesis using the Pfu DNA polymerase (Agilent Technologies, Santa Clara, CA, USA). Primers for mutagenesis were designed by PrimerX (https://www.bioinformatics.org/primerx/, accessed in 30 May 2020). All primers are listed in (Supplementary Table S1). Plasmids were purified using the Plasmid Plus Midi kit (Qiagen, Hilden, Germany).

Prdx1 and Prdx2 WT constructs for recombinant protein expression in *E. coli* (pET17-Prdx1 and pET17-Prdx2), which have already been used in Prdx structural studies [43,44], were kindly provided by the lab of Todd Lowther (Wake Forest School of Medicine, Winston-Salem, NC, USA). The ASK1 thioredoxin-binding domain (ASK1-TBD) (codon-optimized for *E. coli*) was expressed using a pEH vector harboring an N-terminal 6x-His tag along with a maltose binding protein (MBP) and tobacco etch virus (TEV) cleavage site. This construct was purchased from the VIB protein core (Gent University, Gent, Belgium).

All constructs were confirmed by Sanger sequencing.

### 2.4. Transfection of Cell Lines

A total of 2 × 10^5^ HEK293 MSR or AnxA2 KO HEK293 MSR cells were seeded per well of a 12-well plate. The next day, cells were co-transfected with two plasmids (either mLumin-ASK1 or mLumin-STAT3 with mLumin-Prdx (Prdx1 WT, Prdx1 C52A, Prdx1 C173A, or Prdx2 WT)) in equimolar ratio. Transfection was performed using the Lipofectamine 3000 kit (Thermo Fisher Scientific, Waltham, WA, USA) following the protocol provided by the manufacturer. A GFP-encoding plasmid was also co-transfected for normalization purposes in an equimolar ratio with the two plasmids above.

### 2.5. Bimolecular Fluorescent Complementation Assay and Image Analysis

24 h after transfection, cells were treated with either a mixture of xanthine/xanthine oxidase (8 µM of xanthine and 1 mU/mL of xanthine oxidase) (X/XO), 0.8 µM auranofin for 18 h, or a bolus of 100 µM H_2_O_2_ for 30 min. All the oxidants were prepared in DMEM supplemented with 10% FBS and 50 units/mL P/S and this oxidant-containing DMEM then replaced the DMEM cells were growing in (total volume: 1 mL). As each well contained 0.4 × 10^6^ cells, we estimate that in case of 100 µM H_2_O_2_, each cell was exposed to 250 femtomoles of H_2_O_2_. The amount of H_2_O_2_ each cell experienced upon treatment with X/XO and auranofin is more difficult to estimate.

18 h after treatment with X/XO or auranofin, or after 30 min of H_2_O_2_ treatment, mLumin fluorescence and GFP fluorescence images were captured using a fluorescence microscope (Leica DMi8, Wetzlar, Germany), using the 10× (506406) objective. Samples were excited with the 561 nm (for mLumin) and 488 nm (for GFP) laser lines and fluorescence was detected with a 600–680 nm filter for mLumin, and 500–580 nm for GFP.

All images were captured in RAW format and the mLumin and GFP fluorescence-integrated intensities (i.e., the sum of all the pixel values with intensities in a certain threshold selected to avoid saturation) were determined using ImageJ (https://imagej.nih.gov/ij/, accessed in 30 July 2020). The final readout (mLumin intensity normalized to GFP intensity) was obtained by dividing the integrated intensity of mLumin by that of GFP in the same threshold range.

### 2.6. Validation of Bimolecular Fluorescent Complementation Construct Expression

A total of 4.8 × 10^5^ HEK293 MSR or AnxA2 KO HEK293 MSR cells were seeded per well of a 6-well plate. After 24 h, cells were co-transfected with two plasmids encoding the mLumin constructs as described above. Co-transfected cells were harvested after 48 h, pelleted by centrifugation at 4500× *g* for 6 min at 4 °C, resuspended in lysis buffer (20 mM Tris/HCl, pH 7.4, cOmplete™ EDTA-free Protease Inhibitor Cocktail (Roche, Basel, Switzerland), 0.1 mM EDTA, 0.5 mM AEBSF, and 0.5 mM MgCl_2_), and rotated for 30 min at 4 °C, and the lysate was clarified by centrifuging at 16,000× *g* for 20 min at 4 °C. Protein concentration was determined using the Bio-Rad Protein assay (Cat. 500-0006, Bio-Rad Laboratories, Richmond, VA, USA).

The protein samples were separated by SDS-PAGE and transferred to polyvinyl difluoride (PVDF) membranes (Immobilon-P, Millipore, Burlington, MA, USA) using the Trans-Blot^®^ Turbo^TM^ transfer system (Bio-Rad Laboratories, Richmond, VA, USA). Membranes were probed with appropriate antibodies and visualized by the alkaline phosphatase substrate 5-bromo-4-chloro-3-indolyl phosphate p-nitroblue tetrazolium chloride (BCIP/NBT) (Abcam, Cambridge, UK).

### 2.7. Purification of Recombinant Proteins

All pellets of recombinant proteins in this paper were prepared following the same protocol unless stated otherwise. All proteins were expressed in the BL21 *E. coli* strain (New England Biolabs, Ipswich, MA, USA).

### 2.8. Purification of Recombinant Wild-Type Peroxiredoxin-1 (Prdx1 WT) and Prdx1 Mutants (Prdx1 C52A and Prdx1 C173A)

For expression of the Prdx1 WT and mutant constructs, 1 L LB media with ampicillin (100 μg/mL) was inoculated with a 100-fold dilution of an overnight pre-culture and grown at 37 °C with shaking at 120 rpm until the exponential growth phase was reached (OD_600_ = 0.4−0.6), then cooled to 16 °C, induced with 0.4 mM isopropyl-β-D-1-thiogalactopyranoside (IPTG), and further grown overnight at 16 °C with shaking at 120 rpm.

The purification of Prdx1 WT and Prdx1 mutants (C52A and C173A) were performed following the same protocol. Cells were pelleted by centrifugation at 6150 × *g* for 15 min at 4 °C and resuspended in lysis buffer (50 mM HEPES/NaOH, pH 7.4, 250 mM NaCl, 0.1 mg/mL AEBSF, 1 µg/mL Leupeptin, 50 µg/mL DNaseI, and 20 mM MgCl_2_), lysed in a cell cracker at 20 kPsi at 4 °C, and the lysate was clarified by centrifuging at 39,846 × *g* for 30 min at 4 °C. Then, 20% of ammonium sulfate was added to the collected supernatant and centrifuged again at 39,846 × *g* for 30 min at 4 °C to remove the precipitated proteins. After that, the supernatant was passed through a 0.45 µM filter and loaded onto a Phenyl Sepharose^®^ column (Cytiva, Marlborough, MA, USA) equilibrated with binding buffer (50 mM HEPES/NaOH, pH 7.4, 0.1 mM ethylenediaminetetraacetic acid disodium salt (EDTA), 20% ammonium sulfate, 2 mM dithiothreitol (DTT)). The unbound material was removed by washing the resin with 10 column volumes of binding buffer, after which the bound protein was gradient eluted with 10 column volumes of binding buffer without ammonium sulfate. The fractions containing Prdx1 (determined by running on SDS-PAGE gel in both non-reducing and reducing conditions (50 mM DTT)) were collected and dialyzed overnight with 4 buffer changes in the dialysis buffer (50 mM HEPES/NaOH, pH 7.4, 10 mM NaCl, 0.1 mM EDTA, 2 mM DTT) at 4 °C. The dialyzed protein sample was subsequently loaded onto a cation exchange SP Sepharose^®^ Fast Flow column (Cytiva, Marlborough, MA, USA). After the unbound proteins were eliminated by washing with 10 column volumes of the binding buffer with the same composition as the dialysis buffer, the proteins were eluted with a 10 column volumes gradient of elution buffer (binding buffer with 1 M NaCl). The fractions containing Prdx1 were collected and concentrated using a Vivaspin with a 20 kDa cut-off (Sartorius, Göttingen, Germany), and injected onto a size-exclusion Superdex^TM^75 16/600 column (Cytiva, Marlborough, MA, USA) equilibrated with 50 mM HEPES/NaOH, pH 7.4, 150 mM NaCl, 0.1 mM EDTA, 5 mM Tris(2-carboxyethyl)phosphine (TCEP)). Prdx1-containing fractions were collected, concentrated, and the protein concentration was determined spectroscopically using an extinction coefficient of 18,450 M^−1^ cm^−1^. The protein sample was flash-frozen in liquid nitrogen and stored at −80 °C.

### 2.9. Purification of Recombinant Wild-Type Prdx2 (Prdx2 WT)

Cultures of cells expressing Prdx2 WT were grown as described above for Prdx1. Cells were pelleted by centrifugation at 6150× *g* for 15 min at 4 °C and resuspended in lysis buffer (20 mM HEPES/NaOH, pH 7.4, 100 mM NaCl, 0.1 mM EDTA), lysed in a cell cracker at 20 kPsi at 4 °C, and the lysate was clarified by centrifuging at 39,846× *g* for 30 min at 4 °C. Next, 20% of ammonium sulfate was added to the clarified lysate, followed by centrifugation at 39,846× *g* for 30 min at 4 °C. Then, the collected supernatant was passed through a 0.45 µM filter and loaded onto a Phenyl Sepharose^®^ column (Cytiva, Marlborough, MA, USA) equilibrated with binding buffer (20 mM HEPES/NaOH, pH 6.5, 1 mM EDTA, 20% ammonium sulfate). The unbound material was removed by washing the resin with 10 column volumes of binding buffer, after which the bound protein was gradient eluted with 10 column volumes of elution buffer without ammonium sulfate. The fractions containing Prdx2 (determined by running on SDS-PAGE gel in both non-reducing and reducing conditions (50 mM DTT)) were collected and dialyzed overnight with 4 buffer changes in the dialysis buffer (20 mM Tris/HCl, pH 7.9) at 4 °C. Next, the dialyzed protein sample was loaded onto an anion exchange Q Sepharose^®^ Fast Flow column (Cytiva, Marlborough, MA, USA). After the unbound proteins were eliminated by washing with 10 column volumes of the binding buffer with the same composition as the dialysis buffer, the proteins were gradient eluted with 10 column volumes of elution buffer (binding buffer with 0.5 M NaCl). The fractions containing Prdx2 were collected and dialyzed overnight to 20 mM HEPES/NaOH, pH 7.5 with 4 buffer changes at 4 °C. The dialyzed protein sample was collected and concentrated using a Vivaspin with a 20 kDa cut-off (Sartorius, Göttingen, Germany), and injected onto a size-exclusion Superdex^TM^200 16/600 column (Cytiva, Marlborough, MA, USA) equilibrated with 20 mM HEPES/NaOH, pH 7.5. Prdx2-containing fractions were collected, concentrated, and the protein concentration was determined spectroscopically using an extinction coefficient of 21,555 M^−1^ cm^−1^. The protein was flash-frozen in liquid nitrogen and stored at −80 °C.

### 2.10. Purification of the Recombinant Thioredoxin-Binding-Domain of ASK1 (ASK1-TBD)

The pellet of cells expressing 6xHis-MBP-ASK1-TBD was lysed in 50 mM HEPES/NaOH, pH 7.5, 250 mM NaCl, 0.1 mg/mL AEBSF, 1 µg/mL Leupeptin, 50 µg/mL DNaseI, and 20 mM MgCl_2_ and clarified the same way as Prdx1 and Prdx2. The supernatant was passed through a 0.45 µM filter and loaded onto a Ni^2+^-Sepharose^®^ 6 Fast Flow column (Cytiva, Marlborough, MA, USA) equilibrated with binding buffer containing 50 mM HEPES/NaOH, pH 7.5, 150 mM NaCl, and 20 mM imidazole. The unbound proteins were eliminated by washing the resin with 10 column volumes of binding buffer. The MBP-ASK1-TBD protein was eluted with a linear 10 column volume gradient with elution buffer (50 mM HEPES/NaOH, pH 7.5, 150 mM NaCl, 500 mM imidazole). The fractions containing MBP-ASK1-TBD (determined by SDS-PAGE) were pooled and dialyzed overnight to 50 mM HEPES/NaOH, pH 7.5, 150 mM NaCl, 0.1 mM EDTA, 5 mM DTT with 4 buffer changes at 4 °C. The sample was concentrated using a 20 kDa cut-off Vivaspin concentrator (Sartorius, Göttingen, Germany) and injected onto a size-exclusion Superdex^TM^75 16/600 column (Cytiva, Marlborough, MA, USA) equilibrated with 50 mM HEPES/NaOH, pH 7.5, 150 mM NaCl, 0.1 mM EDTA, 5 mM TCEP. Fractions containing MBP-ASK1-TBD were collected and concentrated using the same Vivaspin 20 kDa cut-off concentrator. The protein concentration was determined spectroscopically using an extinction coefficient of 81,250 M^−1^ cm^−1^. The protein was flash-frozen in liquid nitrogen and stored at −80 °C.

After purification, all recombinant proteins were validated by Western blot. The protein samples were separated by SDS-PAGE and transferred to polyvinyl difluoride (PVDF) membranes (Immobilon-P, Millipore) using Trans-Blot^®^ Turbo^TM^ transfer system (Bio-Rad Laboratories, Richmond, VA, USA). Membranes were probed with appropriate antibodies and visualized by the alkaline phosphatase substrate 5-bromo-4-chloro-3-indolyl phosphate p-nitroblue tetrazolium chloride (BCIP/NBT) (Abcam, Cambridge, UK).

### 2.11. Ferrous Oxidation-Xylenol Orange (FOX) Assay

Prdx1 WT, Prdx1 C52A, and Prdx1 C173A were reduced with 50 mM DTT for 30 min at room temperature. DTT was removed using a Hitrap^®^ desalting column (Cytiva, Marlborough, MA, USA) equilibrated with 50 mM HEPES/NaOH, pH 7.4, 100 mM NaCl. The protein concentration was determined spectroscopically using an extinction coefficient of 18,450 M^−1^ cm^−1^ for Prdx1.

The FOX assay was performed based on the protocol described by Nelson et al. (2011) [45]. Briefly, the FOX reagent was prepared by mixing 1 part of FOX A with 100 parts of FOX B. FOX A consists of 25 mM ammonium ferrous sulfate in 2.5 M sulfuric acid (H_2_SO_4_), and FOX B consists of 100 mM sorbitol and 125 µM xylenol orange. Both FOX A and FOX B solutions were prepared in water.

Prior to the assay, the H_2_O_2_ stock concentration was determined by measuring the absorbance at 240 nm (ε_240_ = 43.6 M^−1^ cm^−1^). To quantify the H_2_O_2_ concentrations, a calibration curve of increasing concentrations of H_2_O_2_ (0 µM to 250 µM) was created. For this purpose, 10 µL of each H_2_O_2_ solution was mixed with 490 µL FOX reagent, vortexed, and incubated in the dark for 30 min. 200 µL of the colorimetric solution was then transferred to the microtiter clear 96-well plate (Thermo Fisher Scientific) and the absorbance was measured at 560 nm using the Spectramax 340PC microplate reader (Molecular Devices, San Jose, CA, USA). Absorbance was plotted vs. concentration. The data were fitted with a linear regression and the R^2^ was determined.

Protein samples were mixed with 200 µM H_2_O_2_ and 1 µM of DTT at a final protein concentration of 2 µM. At time-points 1, 2, 5, 7, and 10 min, 10 µL of the protein-H_2_O_2_ sample was mixed with 490 µL FOX reagent, vortexed, and incubated in the dark for 30 min. Then, 200 µL of the colorimetric solution were transferred to the microtiter clear 96-well plate and absorbance was measured at 560 nm. The concentration of the remaining H_2_O_2_ in the sample was interpolated using the regression equation from the calibration curve of H_2_O_2_.

### 2.12. Horseradish Peroxidase (HRP) Assay for Determination of k_SOH_

The second-order rate constant of the pre-reduced Prdx recombinant proteins (WT and C173A) was determined to confirm the expected active function. For this, the HRP assay was used to monitor the Prdx’s ability to compete with HRP in the reduction of H_2_O_2_, as described [45]. Briefly, in 96-well UV plates, in a total volume of 150 µL, six to eight different protein concentrations of purified Prdx (2–30 µM) were mixed with HRP and H_2_O_2_ to obtain 15 µM HRP (Sigma-Aldrich, St. Louis, MO, USA) and 3 µM H_2_O_2_ final concentrations and the absorbance was measured before and within 60 s of the start of the reaction. HRP forms compound I upon reaction with H_2_O_2_, which can be monitored as a decrease in absorbance at 403 nm [46]. When Prdx outcompetes HRP for the H_2_O_2_, the decrease in absorbance is less because less compound 1 is formed. Thus, data collected at different Prdx concentrations can be transformed to k_SOH_ by plotting the change in absorbance against Prdx concentration. This relationship is represented by this equation:

(Δ*A_max_*−Δ*A_obs_*)/Δ*A_obs_* = *k_SOH_[Prdx]/k_HRP_[HRP]*

Experiments were done in triplicate for each Prdx concentration and repeated with fresh aliquots of enzymes.

### 2.13. Bilayer Interferometry (BLI)

For the BLI assay on Octet^Red^ 96 (ForteBio, Fremont, CA, USA), proteins were reduced with 20 mM DTT for 30 min at room temperature. DTT was removed using a Hitrap^®^ desalting column (Cytiva, Marlborough, MA, USA) equilibrated in 25 mM Tris/HCl, pH 7.4, 25 mM NaCl. To block all free Cys, Prdxs, MBP-ASK1-TBD, and MBP were treated with 20 mM iodoacetamide (IAM) at room temperature for 30 min, and excess IAM was removed on Bio-spin columns (Bio-Rad Laboratories, Richmond, VA, USA). Next, the number of free thiols was determined using Ellman’s reagent (5,5-dithio-bis-(2-nitrobenzoic acid)) (DTNB assay) to confirm that all potential thiol groups were blocked [47].

The protein MBP was used as a reference (negative control) to eliminate the binding possibility of Prdx1 WT to the MBP part of the MBP-ASK1 fusion protein, and Prdx2 WT was used to determine the selectivity of the Prdx1:MBP-ASK1-TBD interaction. All proteins were prepared in 25 mM Tris/HCl, pH 7.4, 25 mM NaCl, 1% bovine serum albumin (BSA), 0.05% Tween 20, and 0.01 mM maltose. His-tagged MBP-ASK1-TBD and His-tagged MBP were loaded onto the Ni^2+^-NTA sensors (ForteBio, Fremont, CA, USA) at a concentration of 0.45 µM and 0.15 µM, respectively. The concentration of analytes (Prdx1 WT, Prdx1 C52A, Prdx1 C173A, and Prdx2 WT) was fixed at 1 µM. The assay was done in the presence of 10 µM DTT for the reducing condition and 10 µM H_2_O_2_ for the oxidizing condition. Data were obtained with the Data Acquisition 9.0 (ForteBio, Fremont, CA, USA) software of the instrument. To calculate association (*k_on_*) and dissociation rate (*k_off_*) constants, different concentrations of the analyte (Prdx1s) were utilized, and the data were analyzed by the Data Analysis 9.0 software (ForteBio, Fremont, CA, USA). In this software, the association curves were fitted through the equation:

$$y=R_{max}\frac{1}{1+\frac{k_{off}}{k_{on}\times \left[analyte\right]}}\left(1-e^{-\left(k_{on}\times \left[analyte\right]+k_{off}\right)t}\right)$$

The dissociation curves were fitted through the following equations:

$$y=y_{0}\;e^{-k_{off}\left(t-t_{0}\right)}$$

$$y_{0}=R_{max}\frac{1}{1+\frac{k_{off}}{k_{on}\times \left[analyte\right]}}\left(1-e^{-\left(k_{on}\times \left[analyte\right]+k_{off}\right)t_{0}}\right)$$

where *y* is the BLI signal (in nm), indicating the level of binding as nm shift, while *y*_0_ represents the nm shift at the beginning of the dissociation phase, *t* is the time (s), and *t*_0_ is the time at the beginning of the dissociation phase. [*Analyte*] is the given concentration of Prdx1 or its variants, and *R_max_* is the fitted maximum binding of the analyte to a given immobilized ligand on the biosensor surface.

### 2.14. Circular Dichroism (CD)

The purified Prdxs (WT, C52A, and C173A) were pre-reduced with 20 mM DTT at room temperature for 30 min, and excess DTT was subsequently removed using a Hitrap^®^ desalting column (Cytiva, Marlborough, MA, USA) equilibrated with 10 mM potassium phosphate buffer pH 7.4, 100 mM potassium fluoride (KF). The protein concentrations were determined spectrophotometrically, using an extinction coefficient of 18,450 M^−1^ cm^−1^. Then, the Prdxs were treated with H_2_O_2_ at 1/10 molar ratio of Prdx/H_2_O_2_ for 30 min at room temperature. Excess H_2_O_2_ was removed using Bio-spin columns (Bio-Rad Laboratories, Richmond, VA, USA) following the user manual and then Prdxs were concentrated to 0.1 mg/mL and 0.05 mg/mL for the reduced and oxidized samples, respectively. The CD spectrum (190–260 nm) was recorded using the CD Spectrometer J-175 (Jasco, Tokyo, Japan). The molar ellipticity [*θ*] was calculated using the equation:

$$\left[\theta \right]=\frac{\theta \times M}{C\times l\times n}$$

where *θ* is the ellipticity in degree, *M* is the molecular mass, *C* is the concentration of protein in mg/mL, *l* is the pathlength in cm, and *n* is the number of residues of the protein.

### 2.15. Data Analysis

Graphpad Prism (GraphPadSoftware, version 9.1.0. San Diego, CA, USA) was used for statistical analysis of the data. The statistical method used was two-way ANOVA and Dunnett’s multiple comparison test. The data are displayed as mean ± SD. A minimum significant level of *p* ≤ 0.05 was set.

## 3. Results

### 3.1. In Silico Modeling and Docking Predicts That Prdx1-LC and ASK1-LN Form the Most Stable Combination

**Figure 2:**
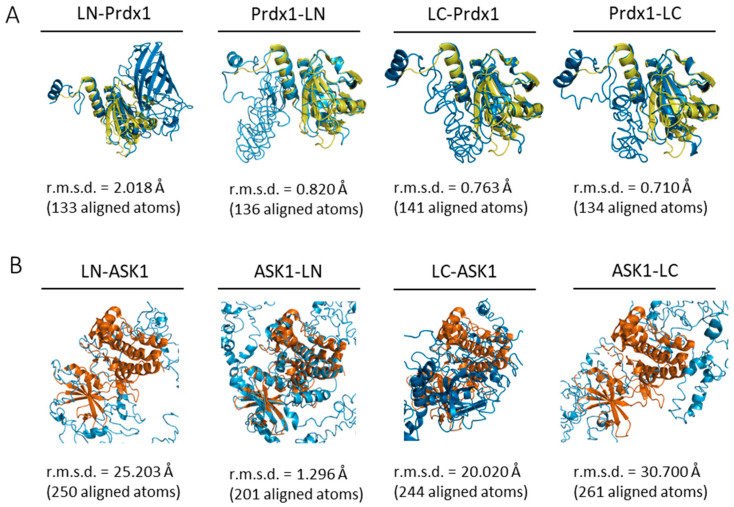
All ab initio Prdx1-mLumin models and the ASK1-LN model maintain the structural fold of Prdx1 and ASK1. Prdx1- and ASK1-mLumin ab initio models were superimposed with the human Prdx1 C83S mutant (PDB ID: 4XCS) andthe human ASK1 kinase domain (PDB ID: 2CLQ). (**A**) All Prdx1-mLumin ab initio models (blue) showed a low r.m.s.d. value with human Prdx1 C83S mutant (PDB: 4XCS) (yellow). (**B**) Only the ASK1-LN ab initio model (blue) showed a superimposition with a relatively low r.m.s.d. value with human ASK1 kinase domain (PDB ID: 2CLQ) (orange). The r.m.s.d. value is displayed for each superimposition. Superimpositions were done in PyMol (Version 2.4.1, Schrödinger, Inc., New York, USA) [40]. LN: large N-terminal half of mLumin. LC: small C-terminal half of mLumin.

The first aim of this study was to confirm the Prdx1:ASK1 interaction reported by Jarvis et al. (2012) [14] where the interaction was detected by co-immunoprecipitation on cell lysates. For this purpose, we chose to use a bimolecular fluorescence complementation assay (BiFC) with mLumin. As with any BiFC assay, the two fragments of the mLumin β-barrel can be attached to either the N- or C-terminus of the two putatively interacting proteins, resulting in 8 possible constructs (4 for each protein), leading to 8 possible BiFC assay combinations [48]. To identify the most stable mLumin fragment-protein combination for Prdx1 and ASK1, we decided to follow an in silico approach. First, the I-TASSER webserver [37] was used to build the 8 possible mLumin-Prdx1 and -ASK1 fusion protein 3D structures (Table 1). The confidence and reliability of the resulting 3D models were evaluated by two parameters: the confidence-score (C-score, between −5 and 2) and the estimated template modeling-score (TM-score). A higher C-score means higher confidence and a cut-off of −1.5 is used to select models of correct topology. A TM-score equal to 1 indicates two identical structures and a TM-score lower than 0.17 indicates random structure pairs. A TM-score greater than 0.5 indicates two structures with the same folding. Though our results fall outside of these values (Table 1), they are acceptable given the synthetic nature of our proteins, therefore we did not discard any of our models. Next, we examined whether the secondary structure of the models was maintained by superimposing them with already solved structures of Prdx1 (PDB ID: 4XCS) and of the ASK1 kinase domain (PDB ID: 2CLQ) in PyMol (Version 2.4.1, Schrödinger, Inc., New York, NY, USA) [40] (Figure 2), where a low root mean square deviation (r.m.s.d.) was indicative of an unperturbed structure. Our results suggest that while the Prdx1 structure is maintained in all four possible mLumin fragment-Prdx1 combinations, the ASK1 structure is only undisrupted in the ASK1-LN fusion model.

Therefore, the only 2 possible BiFC combinations were ASK1-LN with either LC-Prdx1 or Prdx1-LC. To find out which of the two combinations was the most stable one, we performed docking simulations using the HADDOCK server [41]. The stability of the predicted dockings (Table 2) was judged by the HADDOCK score—a linear combination of various energies and buried surface area, with a lower HADDOCK score indicating a more stable docking.

$$HADDOCK\;score=1.0\times E_{van\;der\;Waals}+0.2\times E_{electrotastic}+1.0\times E_{desolvation}+0.1\times E_{AIR}$$

Based on the HADDOCK score, the most stable docking was when ASK1-LN interacted with Prdx1-LC, which presented a HADDOCK score of −362.1 ± 12.6 (Table 2).

In addition, the reliability of the predicted dockings can be evaluated by the Z-score (Table 2). The Z-score indicates how many standard deviations from the HADDOCK score average the cluster was located, with a more negative Z-score indicating a more reliable docking. Here, the docking between ASK1-LN and Prdx1-LC presented the lower Z-score. This further confirmed that the ASK1-LN—Prdx1-LC will yield the most stable and reliable docking in BiFC [41].

We subsequently used the ASK1-LN and Prdx1-LC constructs to test the interaction of Prdx1 and Prdx1 mutants with ASK1 in HEK293 MSR cells. Incidentally, similar constructs were used by Talwar et al. (2020) in the study of the Prdx2:STAT3 interaction by BiFC—STAT3-LN and Prdx2-LC [35]. For this reason, for the Prdx2 construct which we used in this study to assess the specificity of the interaction for Prdx1, the LC fragment of mLumin was fused to the C-terminus of Prdx2.

### 3.2. Prdx1 and Prdx2 Interact with ASK1 in Live HEK293 MSR Cells in an H_2_O_2_-Dependent Manner

**Figure 3:**
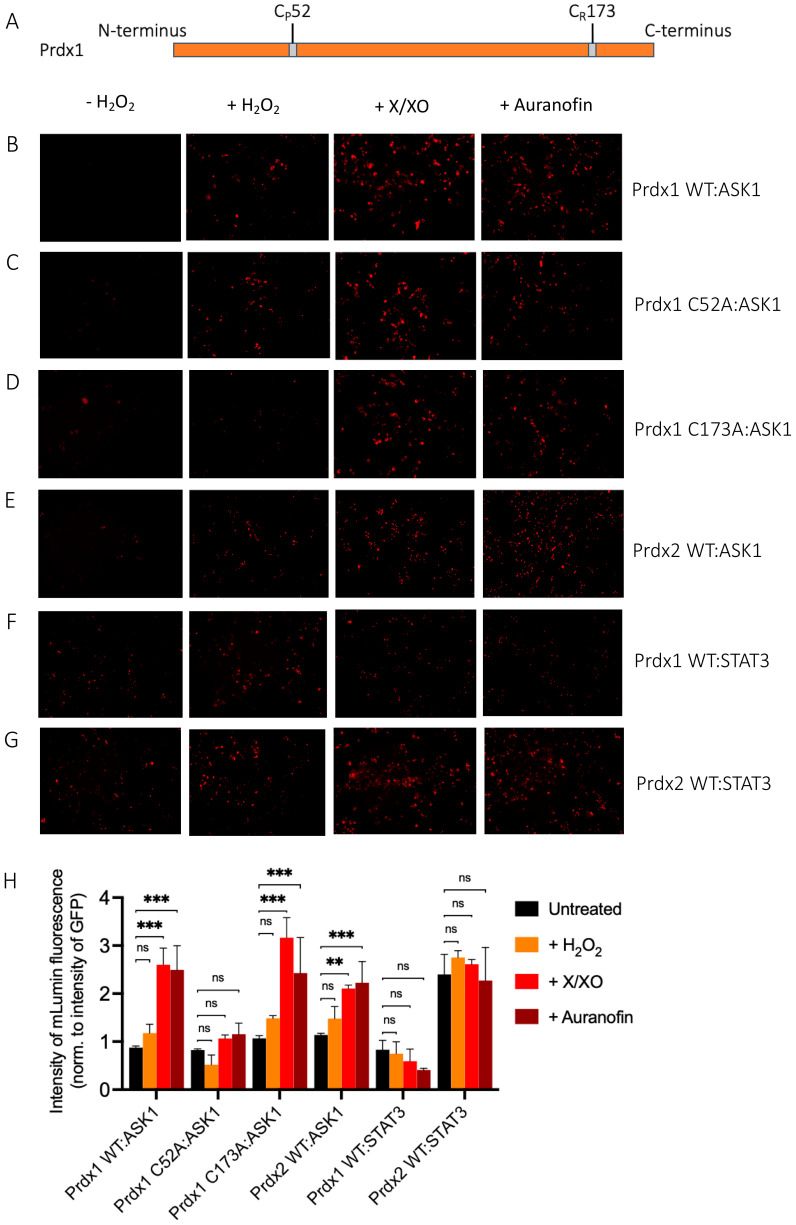
Prdx1 (WT, C52A, and C173A) and Prdx2 WT interact with ASK1 in HEK293 MSR. The fluorescent signal (red dots) emitted by the mLumin protein upon protein complementation (i.e., upon the interaction of the ASK1-LN and Prdx1-LC proteins) in live HEK293 MSR cells is shown. (**A**) The locations of critical cysteine residues of Prdx1. BiFC signal upon (**B**) Prdx1 WT:ASK1, (**C**) Prdx1 C52A:ASK1, (**D**) Prdx1 C173A:ASK1, (**E**) Prdx2 WT:ASK1, (**F**) Prdx1 WT:STAT3, and (**G**) Prdx2 WT:STAT3 interaction. The treatments from left to right were: no treatment, 100 µM H_2_O_2_ for 30 min, X/XO (8 µM X and 1 mU/mL XO) for 18 h, and 0.8 µM auranofin for 18 h. (**H**) Quantified mLumin/GFP values in HEK293 MSR cells (from Figure 3B–G); Prdx1 WT:STAT3 and Prdx2 WT:STAT3 were the respective negative and positive controls. All images were captured with the total magnification of 100×. The GFP images used for normalization are presented in Figure S2. The results are representative of *n* = 3 independent experiments. All bar charts in this figure represent the mean ± SD. ** *p* ≤ 0.01; *** *p* ≤ 0.001; ns not significant; based on an unpaired ANOVA test.

The ASK1-LN and Prdx1-LC constructs selected from our in silico modeling were transiently co-transfected into HEK293 MSR cells. An expression construct for GFP was co-transfected as a third plasmid for normalization purposes. Apart from the Prdx1 WT, we also performed BiFC on ASK1-LN with the peroxidatic (C52A) and resolving (C173A) mutants of Prdx1-LC (Figure 3A). We used several ways to increase intracellular H_2_O_2_ levels, which differed by the duration of the treatment and the place of H_2_O_2_ generation/application. These were a short bolus of 100 μΜ H_2_O_2_ for 30 min, a combination of xanthine/xanthine oxidase (X/XO) for 18 h, which generated extracellular H_2_O_2_ that then entered cells via aquaporins, and the thioredoxin reductase inhibitor auranofin (also for 18 h). As the thioredoxin system reduces peroxiredoxins, its inhibition would inevitably lead to a build-up of oxidized peroxiredoxins, and therefore, an accumulation of endogenous H_2_O_2_. We employed these longer treatments, as treatment of cells with bolus doses of peroxide could result in artifactual oxidation. As can be seen in Figure 3B, in the absence of H_2_O_2_, we observed no fluorescence, whereas all three conditions that lead to an increase in H_2_O_2_ levels led to substantial fluorescence complementation with the longer treatments (X/XO and auranofin) resulting in up to 3-fold higher fluorescence than a bolus of H_2_O_2_ (Figure 3B,H). This finding suggests that Prdx1 WT interacts with ASK1 only upon the exposure of cells to H_2_O_2_, unlike the Prdx2:STAT3 interaction, which occurs independently of and prior to exposure to H_2_O_2_ [35].

The Prdx1 C52A and Prdx1 C173A mutants also showed fluorescence complementation upon an increase in intracellular H_2_O_2_ levels, indicating that they also interact with ASK1 (Figure 3C,D,H). However, in case of the former, the fluorescence was 2-fold lower than with Prdx1 WT and C173A, suggesting that the peroxidatic Cys (C52) plays an important role in the Prdx1:ASK1 interaction, yet it can still take place in cells to some extent in its absence. Again, longer oxidant treatments (with X/XO and auranofin) led to higher fluorescence. To ensure that the observed differences were indeed reflecting differences in protein–protein interactions and not expression levels, we assessed the expression levels of all constructs by Western blot (Supplementary Figure S1A) and found no differences between the different Prdx1 mutants. Interestingly, the BiFC signal was also observed between Prdx2 WT and ASK1, demonstrating that Prdx2 also interacts with ASK1 in an H_2_O_2_-dependent manner. While the same level of fluorescence complementation was observed for the Prdx2 WT:ASK1 interaction as for Prdx1 WT upon treatment with a bolus of H_2_O_2_, treatment with X/XO and auranofin did not increase the signal to the same extent as observed with Prdx1 WT and Prdx1 C173A (Figure 3E,H). Our BiFC results also imply that the Prdx2 WT:ASK1 interaction is greater than that of Prdx1 C52A:ASK1. It should be noted, however, that BiFC signal level comparisons with Prdx2:ASK1 should be taken with caution, as our Western blot analysis showed lower ASK1 expression in the Prdx2 sample compared to the Prdx1 ones. Prdx1 WT:STAT3 and Prdx2:STAT3 were employed as the negative and positive controls, respectively (Figure 3F,G). As expected, quantitative analysis revealed that the BiFC signal between Prdx1 WT and STAT3 was lower than for Prdx1 C52A and ASK1, while the Prdx2 WT:STAT3 signal was at a comparable level compared to Prdx1 WT:ASK1 after longer exposure to H_2_O_2_ (Figure 3H).

Taken together, we demonstrated that Prdx1 and ASK1 interact in live cells in an H_2_O_2_-dependent manner and that this interaction requires the peroxidatic Cys of Prdx1. However, to a lesser degree, the interaction can still occur without it. We speculate that this may be due to the presence of an unidentified scaffolding protein that will still bring Prdx1 close to ASK1 for fluorescence complementation to occur (i.e., 10 nm [49]), even if the C52 is mutated and Prdx1 is unable to participate in a productive redox-relay. Intriguingly, our results also show that Prdx2 interacts with ASK1 to some extent, confirming the findings of Stöcker et al. [16], who reported this interaction using a pull-down experiment with a tagged Prdx1 [16]. Similarly to us, in that study, the authors observed that Prdx1 interacts with ASK1 only upon treatment with at least 10 μΜ H_2_O_2_ for 1 min. By contrast, Prdx2 co-immunoprecipitated with ASK1 even without the addition of exogenous H_2_O_2_.

### 3.3. Prdx1 Interacts with the Thioredoxin Binding Domain of ASK1 In Vitro

Next, we addressed the question of whether Prdx1 interacts with ASK1 directly or whether the presence of a scaffolding protein is required. To this end, we recombinantly expressed and purified Prdx1 and the thioredoxin binding domain of ASK1 (ASK1-TBD). We focused on the thioredoxin binding domain of ASK1, as it contains the redox-sensitive Cys residue (C250) and has been shown to be redox-regulated and involved in the interaction with Prdx1 [50]. While we could successfully purify Prdx1 and its variants (Supplementary Figure S3), the purification of ASK1-TBD proved to be a challenge, as the protein was expressed in inclusion bodies, which drastically affected the purification yield. For this reason, we decided to conjugate the maltose binding protein tag (MBP) to the N-terminus of ASK1-TBD to increase the solubility, and therefore the yield. This approach was proven to be effective since we could purify ASK1-TBD with an MBP tag (MBP-ASK1-TBD) with a high yield (Supplementary Figure S3).

As a quality control of the recombinant Prdx1 variants, we assessed their peroxidase activity in a FOX assay with DTT as electron donor (Supplementary Figure S4). Prdx1 WT and the resolving Cys mutant Prdx1 C173A showed peroxidase activity by consuming H_2_O_2_. As expected, the peroxidatic mutant Prdx1 C52A showed almost no activity, while the most active form was Prdx1 C173A. This Prdx1 variant was less sensitive to hyperoxidation compared to Prdx1 WT. Peskin et al. [51] also noticed this in some of the resolving cysteine mutants of Prdx2, where they showed that the rate of hyperoxidation was dramatically decreased compared to Prdx2 WT [51].

The two active Prdx1 variants, WT and C173A, were verified further using HRP assay to calculate *k*_SOH_. Both enzymes showed rates in the expected range of 10^7^ M^−1^ s^−1^, with Prdx1 WT having a *k*_SOH_ of 1.3 × 10^7^ ± 0.3 M^−1^ s^−1^, and Prdx1 C173A having a *k*_SOH_ of 1.7 × 10^7^ ± 0.2 M^−1^ s^−1^. The benefit of activity confirmation by this additional method is the ability to use low H_2_O_2_ concentrations (3 µM), thus avoiding confounding variables like competing inactivation via hyperoxidation that can sometimes occur in a FOX assay, where higher H_2_O_2_ concentrations (200 µM) are used.

**Figure 4:**
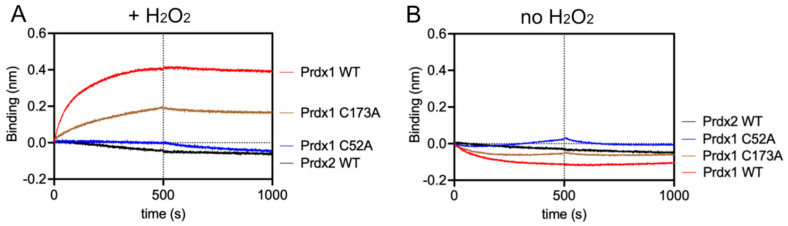
Prdx1 interacts with ASK1-TBD in the presence of H_2_O_2_ through its peroxidatic cysteine via disulfide bond formation, while Prdx2 does not interact with ASK1-TBD. (**A**) BLI assay in the presence of 10 µM H_2_O_2_. (**B**) BLI assay in the absence of H_2_O_2_ (to guarantee no oxidation, 10 µM DTT was added to the buffer solution). The concentration of Prdx1 WT, C52A, C173A, and Prdx2 WT were fixed at 1 µM and MBP was used as reference. The *y*-axis indicates the wavelength shift (in nm) of the Octet^Red^ 96 instrument. The vertical dashed line separates the association and dissociation phases.

After protein purification and Prdx quality assessment, we studied the binding kinetics of the Prdx1:ASK1-TBD interaction by bio-layer interferometry (BLI). Specifically, we determined the k_on_ and k_off_ values of Prdx1 WT and mutants to ASK1-TBD. Experimentally, MBP-ASK1-TBD was tethered to the sensor-tip via its 6x-His tag and the different Prdx1 variants were added at increasing concentrations in the presence of 10 µM H_2_O_2_. As can be seen in Table 3, Figure 4A, and Supplementary Figure S5A,B, in these conditions, Prdx1 WT and Prdx1 C173A interacted with ASK1-TBD, while no interaction was seen for Prdx1 C52A or Prdx2, contrary to our observations in cells (Figure 3C,E). It should be noted, however, that the absence of a resolving cysteine (C173) also affected the interaction, as it had a slightly lower k_on_ value (Table 3). To rule out the possibility that these differences in the interaction with ASK1-TBD were due to possible structural distortions caused by the C173A and C52A mutations in Prdx1, we assessed the secondary structure of Prdx1 WT and its variants by circular dichroism (CD) in both reduced and oxidized states (Supplementary Figure S6A,B). In both cases, the secondary structure of the two Prdx1 mutants was maintained and similar spectra to the Prdx1 WT were obtained.

To demonstrate that the interaction depends on the formation of a mixed disulfide, we repeated the BLI assay (i) in the absence of H_2_O_2_ (Figure 4B), as well as (ii) with all cysteines of either ASK1-TBD or Prdxs (WT, C52A, and C173A) alkylated with IAM (to guarantee complete alkylation, the number of free Cys was checked with a DTNB assay) (Supplementary Figure S5C). In both cases, no association curves could be recorded, indicating that the environmental structure of the peroxidatic Cys is required for the interaction via a mixed disulfide bond and supporting our conclusion that the Prdx1:ASK1-TBD interaction relies on mixed disulfide formation. Additionally, the extremely slow k_off_ values in the absence of reducing agent clearly suggest disulfide bond formation (Table 3).

### 3.4. The Prdx2:ASK1 Interaction Does Not Depend on AnxA2

**Figure 5:**
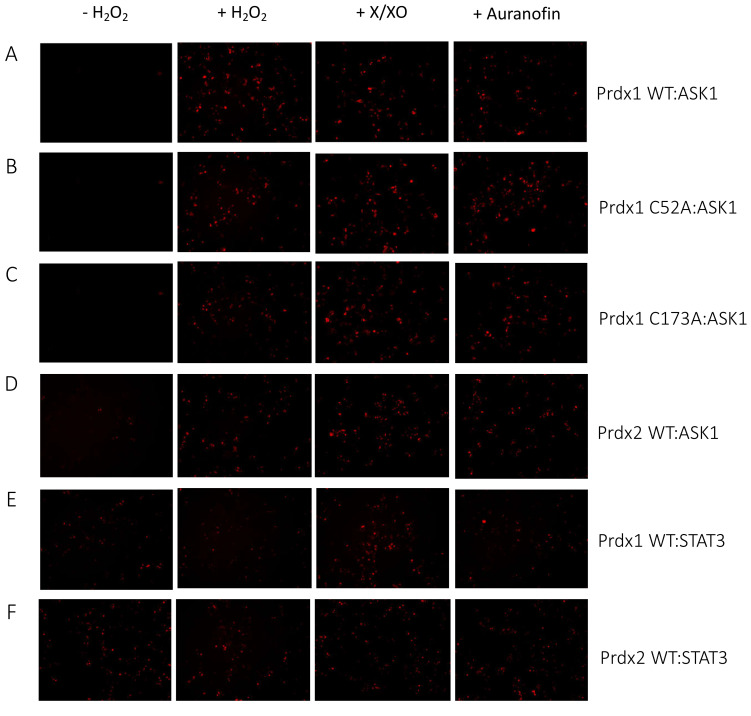

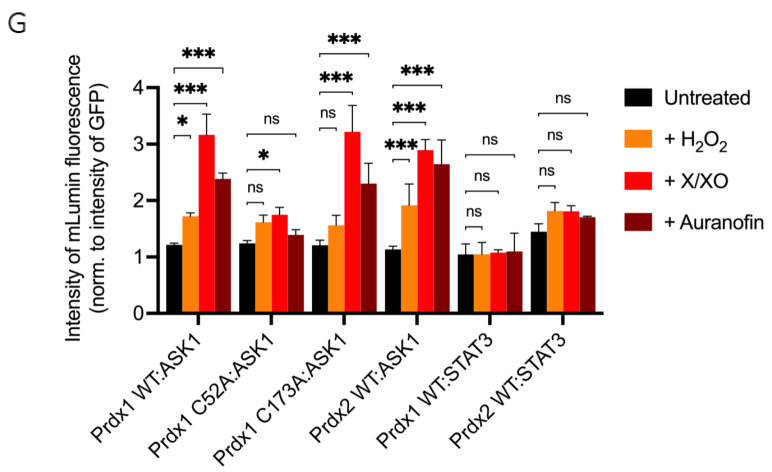
Prdx1 (WT, C52A, and C173A) and Prdx2 WT interact with ASK1 independently of AnxA2. The fluorescent signal (red dots) emitted by the mLumin protein upon proteins complementation in AnxA2 KO HEK293 MSR cells (i.e., the interaction of the ASK1-LN with Prdx1-LC) is shown. BiFC signal upon (**A**) Prdx1 WT:ASK1, (**B**) Prdx1 C52A:ASK1, (**C**) Prdx1 C173A:ASK1, (**D**) Prdx2 WT:ASK1, (**E**) Prdx1 WT:STAT3, and (**F**) Prdx2 WT:STAT3 interaction. The treatments from left to right were: no treatment, 100 µM H_2_O_2_ for 30 min, X/XO (8 µM X and 1 mU/mL XO) for 18 h, and 0.8 µM auranofin for 18 h. (**G**) Quantified mLumin/GFP values in AnxA2 KO HEK293 MSR cells; Prdx1 WT:STAT3 and Prdx2 WT:STAT3 were the controls. All images were captured with the total magnification of 100×. The GFP images used for normalization are presented in Figure S7. The results are representative of *n* = 3 independent experiments. All bar charts in this figure represent the mean ± SD. * *p* ≤ 0.05; *** *p* ≤ 0.001; ns not significant; based on an unpaired ANOVA test.

As we observed no interaction between Prdx2 and ASK1 in vitro (Figure 4) yet our BiFC assay results showed that they do interact in cells (Figure 3E), we next asked whether such stark differences could be explained by an absence of the facilitator. Indeed, the search for a facilitator for the Prdx2:STAT3 interaction was triggered by similar observations, where a Prdx2:STAT3 interaction was observed in cells yet not in vitro on purified proteins. As it is known that the Prdx2:STAT3 interaction depends on AnxA2, we decided to test whether the Prdx2:ASK1 interaction is also AnxA2-dependent [35]. Further, even though our in vitro results (Figure 4) indicated that the Prdx1:ASK1 interaction does not strictly require a facilitator, we nevertheless also tested whether AnxA2 plays a role in mediating this interaction. To this end, we repeated the BiFC assay in HEK293 MSR AnxA2-knockout cells. As can be seen in Figure 5, the Prdx1 WT and Prdx2 WT interaction with ASK1 were AnxA2-independent. This contrasts with the Prdx2 and STAT3 interaction, which was diminished in the absence of AnxA2 (compare with Figure 3H). As in the BiFC assay in WT cells, expression levels of the different Prdx1 variants and Prdx2 were assessed by Western blot to make sure that the observed differences in fluorescent complementation could not be accounted for by differences in expression levels (Supplementary Figure S1). These results hint at the need for a facilitator for the Prdx2:ASK1 interaction that is, however, not AnxA2.

## 4. Discussion

We decided to bring the first reported mammalian Prdx redox-relay, Prdx1:ASK1, back into the spotlight. Ever since co-immunoprecipitation experiments on cell lysates showed a putative mixed disulfide between Prdx1 and ASK1 [14], there have been no published attempts to gain more mechanistic insights into this interaction or to at least validate it in intact cells.

Here, we confirmed the Prdx1:ASK1 interaction with an in-cell approach not requiring cell lysis, BiFC, as well as with in vitro experiments using recombinant proteins. We also discovered that Prdx1 and ASK1 interact in the absence of a scaffolding protein. Even though it has been reported that the central regulatory region of ASK1 can serve as a recruitment platform for ASK1 substrates [52], our in vitro results clearly show that the TBD domain alone is sufficient for the Prdx1:ASK1 interaction, thus ruling out the requirement of another domain of ASK1 acting as a scaffolding protein. Further, we revealed that the Prdx1:ASK1 interaction only occurs upon H_2_O_2_ induction and depends on the peroxidatic cysteine (C52) of Prdx1. Most of these aspects are in total contrast to the well-characterized Prdx2:STAT3 interaction, where Prdx2 and STAT3 are known to associate even prior to induction with H_2_O_2_ and require AnxA2 for a productive oxidative transfer [35].

There are several possible reasons for why Prdx2 and STAT3 require a scaffolding protein to interact, whereas Prdx1 and ASK1 do not, some of which could be related to the precise role of a scaffolding protein. On the whole, it is difficult to generalize on the function of scaffolding proteins for redox-relays, as only two such proteins have been reported—apart from the aforementioned AnxA2 [35], Ybp1 mediates the Orp1:Yap1 relay in yeast [36]. One potential role could be driven by kinetic arguments. Once the sulfenic acid is formed on the peroxidatic cysteine (C_P_-OH) of Prdx1 or Prdx2, it could succumb to two fates: (i) it could be attacked by the thiol of the interactor forming a mixed disulfide bond, or (ii) it could condense with the resolving cysteine of Prdx to form an intersubunit C_P_-C_R_, after which the oxidative equivalents would be transferred to the interactor by thiol–disulfide exchange. Mathematical modeling of the Prdx2:STAT3 redox-relay showed that both mechanisms fit the experimental data well [53]. However, the oxidized form of Prdx (both the sulfenic acid and the C_P_-C_R_ intersubunit disulfide) is also competing with the mM concentrations of glutathione in the cytosol (reaction rate 500 M^−1^ s^−1^) [54] or with thioredoxin (2.1 × 10^6^ M^−1^ s^−1^) [55]. Both reactions could outcompete the reaction with the thiol of the interactor, which is estimated to be in the 10^2^ M^−1^ s^−1^ range for STAT3 [53]. With this in mind, the role of the scaffolding protein would consist in shielding the Prdx C_P_-OH or the intersubunit C_P_-C_R_ disulfide from the cellular reducing systems, as suggested for Ybp1 [36]. The rate constants of the condensation step in which the Prdx C_R_ nucleophilically attacks the C_P_-OH differ by almost two orders of magnitude for Prdx1 and Prdx2 (11 s^−1^ and 0.2 s^−1^, respectively) [56]. Hence, for Prdx2, there is an increased chance of this more long-lived C_P_-OH to be attacked by glutathione compared to Prdx1 C_P_-OH, which argues for an increased need of a scaffolding protein for Prdx2. One other unexplored possibility is post-translational modifications that could be extending C_P_-OH lifetime through structural steric changes. Perhaps the different ways (i.e., PTM versus scaffolding protein) are the means to specificity in binding partner and binding process.

A scaffolding protein could also ensure that the interacting cysteines of Prdx and its interactor align properly for an efficient oxidative transfer, as observed for Orp1:Ybp1:Yap1 [36]. Both STAT3 and ASK1 harbor multiple cysteine residues, but the difference may lie in the cellular location of the interacting partners. While the Prdx2:STAT3 interaction takes place at the membranes [35], where the viscosity may render the rearrangements of the interacting proteins to obtain a proper alignment difficult, the Prdx1:ASK1 interaction is expected to take place in the cytosol [57], which has a viscosity like that of pure water. Despite the molecular crowding of the cytosol, which slows down the diffusion of molecules about four-fold compared to water, the diffusion of macromolecules in membranes is still substantially slower [58]. Interestingly, Orp1 and Yap1, which require Ybp1 to interact, or at least one of their pools, are also membrane-bound [13]. Localization could also explain the need for Prdx2 and STAT3 to pre-assemble—due to the increased viscosity at the plasma membrane, the trafficking of the proteins is expected to be hindered; pre-assembly would significantly reduce the time required for the interaction to occur. Indeed, in vitro experiments have shown a clear dependence of the rates of catalyzed reactions and tertiary complex formation on viscosity [59].

As mentioned above, as there have only been two peroxidase redox-relay complexes featuring a scaffolding protein characterized, all reasons on why the Prdx2:STAT3 redox-relay requires a scaffolding protein but the Prdx1:ASK1 relay does not will remain speculative. As such, the function of the Prdx interactor could also dictate the need for a scaffolding protein. Both Yap1 and STAT3 are transcription factors, whereas ASK1 is a kinase. Another kinase, MST1, was found to interact with Prdx1 in vitro, as assessed through a kinase assay, providing one more example of a Prdx:kinase interaction that occurs without a scaffolding protein. However, it is not clear whether Prdx1 and MST form a redox-relay [60]. Whether these differences between ASK1 and STAT3 oxidation would hold true for other kinases and transcription factors, the physiological meaning of this, and where proteins with other functionalities that form redox-relays with Prdxs lie is a subject of future studies.

Our experiments with the peroxidatic (C52A) and resolving (C173A) mutants of Prdx1 resulted in in vitro vs. in-cell differences. This was especially striking for the peroxidatic cysteine mutant, where the interaction was completely abolished in vitro yet could still occur in cells. This demonstrates that the peroxidatic cysteine is essential for the interaction with ASK1 to occur, yet there are mechanisms in cells, most likely driven by endogenous Prdx1 and other proteins (to be discussed in detail below), that still enable the Prdx1 C52A:ASK1 association. It remains to be clarified if these alternative mechanisms result in true, productive redox-relays or if they are more general protein–protein interactions. Of note, the redox-inactive mutant of Prdx2 could still bind STAT3 and compete with wild type Prdx2 [15].

As for the resolving cysteine mutant, while in cells it was indistinguishable from Prdx1 WT, a difference of less than one order of magnitude was observed in vitro. The reason behind this is likely to be purely technical: BiFC simply cannot capture small differences in reaction rates. The slightly slower k_on_ of the resolving cysteine mutant could be a consequence of minor local structural changes by which the efficiency of reactivity of C_P_ is partially being compromised, as reported recently for Prdx2 [51]. The reaction of the resolving cysteine Prdx1 mutant with urate hydroperoxide was also lower than for the WT [61]. The difference in reaction rates with ASK1 between the Prdx1 WT and resolving cysteine mutant that we observed hints to a Prdx1:ASK1 redox-relay that is preferentially formed by thiol–disulfide exchange (i.e., attack of ASK1 Cys250 on the Prdx1 C_P_-C_R_ inter-subunit disulfide bond) rather than on the Prdx1 C_P_-OH sulfenic acid. This would also be in line with the reaction rate of C_P_-OH condensation with C_R_ (11 s^−1^), which is two orders of magnitude faster than for Prdx2. Nevertheless, our in vitro results also clearly indicate that the procession through the C_P_-OH can still occur, as otherwise we would not have observed an association of the Prdx1 C173A mutant with ASK1.

We also found that, unlike the Prdx2:STAT3 interaction, which is very specific for the peroxidase isoform [35], Prdx2 can also interact with ASK1, but only in cells and to a lesser extent than Prdx1. This hints at the requirement of a scaffolding protein that is not AnxA2, perhaps quite expectedly, as ASK1, unlike STAT3 and AnxA2, is not a membrane-associated protein. Interestingly, the behavior of Prdx2 was very similar to that of the Prdx1 peroxidatic cysteine mutant (C52A) in that it could also only interact with ASK1 in cells. This suggests that there is a third protein mediating this interaction, which would bring Prdx1 C52A close enough to ASK1 for mLumin recombination without the actual formation of a Prdx1-ASK1 mixed disulfide, which does not occur according to our BLI results. The C52A BiFC result thus represents the amount of putative facilitation being accomplished by the unknown facilitator. To summarize more accurately and concisely: the Prdx1:ASK1 interaction is likely enhanced when a facilitator is present but is still able to occur without one. Moreover, even when the C52 of Prdx1 is mutated, if the facilitator is present (i.e., in cells), then it will bring Prdx1 in close proximity to ASK1 to encourage disulfide interaction even if chemically it is unable to occur because of the lack of the peroxidatic cysteine.

**Figure 6:**
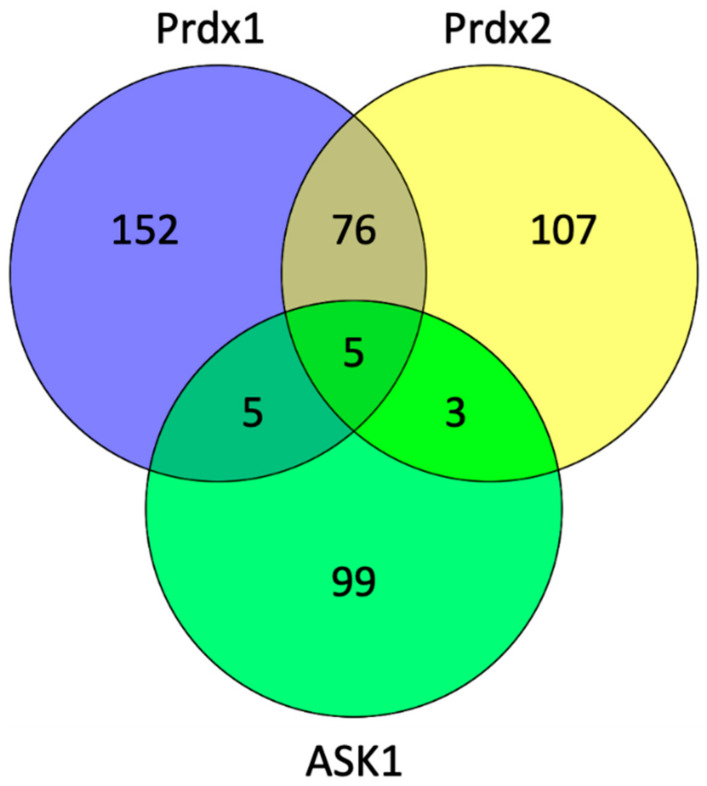
The number of interacting proteins of Prdx1, Prdx2, and ASK1. The diagram was generated by Venny (version 2.1) (https://bioinfogp.cnb.csic.es/tools/venny/, accessed in 30 April 2021) and shows the number of common interactors in the cross sections of the diagram using the data from the PPI database (BioGrid version 4.3.196) (https://thebiogrid.org/, accessed in 30 March 2021) [62].

To explore which proteins can act as potential scaffolding proteins for the Prdx:ASK1 interaction, we searched the BioGrid database (version 4.3.196) to find common interactors between Prdx1, Prdx2, and ASK1 (Figure 6). We found five such interactors, namely, cyclin-dependent kinase 2 (CDK2), egl-9 family hypoxia-inducible factor 3 (EGLN3), glyceraldehyde-3-phosphate dehydrogenase (GAPDH), KIAA1429, and thioredoxin (Trx1).

**Figure 7:**
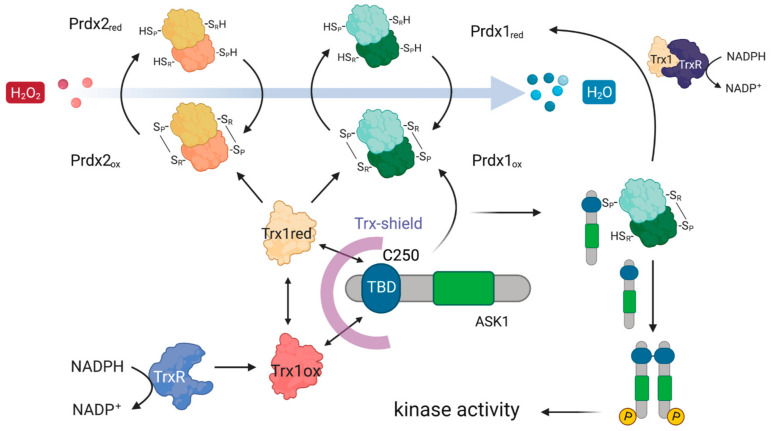
Possible mechanistic model of how Prdx1 and Prdx2 could interact with ASK1. The ASK1-TBD harboring the critical C250 is protected by a Trx-shield. This shield consists of Trx1 which is constantly being recycled from oxidized to reduced Trx1 using electrons from NADPH via TrxR. Upon an increase in H_2_O_2_, Prdx1 and Prdx2 get oxidized and then oxidize Trx1 bound to the ASK1-TBD. As oxidized Trx1 binds the ASK1-TBD with a lower affinity [32], it allows oxidized Prdx1 to outcompete it for binding to the ASK1-TBD, enabling a nucleophilic attack of C250 on the C_P_-C_R_ of Prdx1. Oxidation of ASK1 at C250 leads to ASK1 dimerization and an activation of its kinase activity.

From the five proteins in the overlap, Trx1 is the most likely candidate for the role of a mediator of the Prdx1 and Prdx2 interaction with ASK1, as it fits well with the known role of reduced Trx1 in binding ASK1 and inhibiting its kinase activity at basal H_2_O_2_ levels [32]. Taken together, our results suggest the following model of the Prdx1:ASK1 interaction (Figure 7), which is based on kinetic competition (i) for the binding to the ASK1-TBD: between C_P_-C_R_ Prdx1 (or C_P_-OH of Prdx1) and Trx1 (oxidized and reduced) [32,63] and (ii) for the binding to C_P_-C_R_ Prdx1: between reduced Trx1 and ASK1-TBD. At basal H_2_O_2_ levels, the prevalent species of all the competitors is reduced Trx1, and ASK1-TBD is preferentially bound to it. Upon an increase in H_2_O_2_, Prdx1 and Prdx2 become oxidized (C_P_-C_R_) and are subsequently reduced by Trx1 bound to ASK1. As oxidized Trx1 binds ASK1-TBD with a lower affinity [32] and structural changes in the ASK1-TBD make Trx1 binding unfavorable [64], the chance for the exposed Cys250 of ASK1-TBD to perform a nucleophilic attack on oxidized Prdx1 increases (competition (i)). At the same time, oxidized Trx1 would not compete with Cys250 for C_P_-C_R_ Prdx1 (competition (ii)). In case the ASK1-Prdx1 interaction proceeds via the intermolecular Prdx1 disulfide, this can either come from another Prdx1 dimer or from the other Prdx1 subunit of the Prdx1 dimer that oxidized the ASK1-bound Trx1. According to this model, the Trx1 bound to ASK1 acts as a recruitment platform for Prdx1 (or Prdx2) in the crowded environment of the cell. In vitro, where there is no need to recruit specific proteins, Trx1 is not needed.

The read-out of our BIFC experiments does not allow discrimination between Prdx1 that is oxidizing Trx1 bound to ASK1 or Cys250 of ASK1, as complementation has a distance range up to 10 nm [49]. This would explain why we observe the Prdx2:ASK1 interaction in cells, but not in vitro: fluorescence complementation occurs when Prdx2 is oxidizing Trx1 bound to ASK1 yet does not in fact interact with ASK1 itself. In the case of the Prdx1 peroxidatic cysteine mutant, we can envisage a scenario where it would still bind to Trx1, as was observed for a peroxidatic cysteine mutant of a plant Prdx [65], leading to fluorescence complementation. This interaction occurs exclusively upon treatment with H_2_O_2_, as mutant Prdx1 could still form a dimer with an endogenous Prdx1 (C_P_-OH of the endogenous Prdx1 subunit would condense with the C_R_ of the peroxidatic cysteine mutant) that would oxidize the Trx1. An interaction with Trx1, rather than ASK1 itself, could potentially explain why the Prdx2 and Prdx1 C52A interaction with ASK1 yields lower fluorescence complementation than the Prdx1 WT and the Prdx1 C173A mutant: as the BiFC signal is cumulative, if complementation occurs both when Prdx1 interacts with Trx1 and ASK1, Prdx1 WT would yield higher signals. A repetition of the experiments in Prdx1 KO cells would help to clarify this. Regardless of the precise mechanism, the results of our in vitro data performed without Trx1 suggest that the role of Trx1 is limited to shielding the Cys250 of ASK1 at basal H_2_O_2_ levels and is not necessary for the Prdx1:ASK1 interaction. This Trx-shield is based on an estimated Trx1 concentration in HEK293 cells of 40–50 µM [66] and, taking the K_D_ of reduced (0.3 ± 0.1 μM, [32]) and oxidized (4 ± 2 μM, [67]) Trx1 into account, oxidized Trx1 will directly dissociate from the TBD of ASK1. To see whether Trx1 is needed for ASK1 activation by Prdx1, experiments investigating the kinase activity of ASK1 would be necessary. These, however, are beyond the scope of the present study.

Alternatively, Prdx2 or the Prdx1 peroxidatic cysteine mutant could be interacting with domains other than the TBD, either covalently or non-covalently. To fully understand the Prdx2:ASK1 interaction, a separate study is required featuring various Prdx2 mutants and ASK1 domains using techniques such as co-IP and BiFC.

Another fate of the Prdx1 Cys_P_-OH that was not discussed above is its hyperoxidation to Cys_P_-O_2_H. This sensitivity to hyperoxidation is notably variable between different isoforms. For example, Prdx1 is less prone to hyperoxidation than Prdx2 [44]. The repair of sulfinylated Prdx1 can occur by Srx, which releases the repaired Prdx1 in the SOH form [43]. This opens the possibility of Srx also acting as a scaffolding protein. We envisage that Srx could facilitate the Prdx1-ASK1 interaction both directly and indirectly. Respectively, it could be simply factoring into the amount of SOH available to participate in redox-relays or it could be serving as a scaffold. Moreover, the variability in hyperoxidation sensitivity could be a reason for scaffolding protein requirements for Prdx2 but not Prdx1.

Apart from the insights into the Prdx1:ASK1 interaction, this study also showed that constructs for BiFC can be selected based on an in silico approach using publicly available iTASSER and HADDOCK webservers, thus circumventing the laborious task of testing various combinations of BiFC pairs.

The discovery of AnxA2 as a scaffolding protein for Prdx2:STAT3 [35] was hailed as one that brings us closer to solving the mystery of what dictates the specificity of Prdx redox-relays. The results of the investigation of the Prdx2:CRMP2 interaction then dampened those hopes, as they suggested that the organization of the same redox-relay was cell type-specific [68]. The findings presented here, showing that scaffolding proteins are not needed for mediating all redox-relays, and that Prdx2 can interact with Prdx1 interactors, further suggest that the molecular details of redox-relays are more complex than we anticipated and that we are still a long way away from understanding how Prdxs and their targets find each other in the “molecularly crowded” environment of the cell. The overall role of peroxiredoxins and redox signaling in pathophysiology, however, guarantees that all efforts to disentangle the complexity of redox-relay organization will be fully justified.

## 5. Conclusions

We validated the Prdx1:ASK1 interaction, the first reported redox-relay in mammalian cells, in intact living cells. Our results also revealed that, in contrast to the Prdx2:STAT3 redox-relay, a scaffolding protein is not necessary for the Prdx1:ASK1 interaction, and that it only occurs upon exposure to H_2_O_2_. We also demonstrated that Prdx2 can interact with ASK1, although unlike Prdx1, this interaction seems to require a facilitator that is not AnxA2. Future studies will uncover how specific these differences are for the two Prdx isoforms and for the function of their redox-relay interactors—transcription factor vs. kinase.

## Figures and Tables

**Table 1:** Prdx1-mLumin and ASK1-mLumin fusion proteins do not present random similarity with the templates used for modeling.

N- to C-Terminus	C-Score	Estimated TM-Score
LN-Prdx1	−2.43	0.43 ± 0.14
Prdx1-LN	−2.82	0.39 ± 0.13
LC-Prdx1	−2.65	0.41 ± 0.14
Prdx1-LC	−2.40	0.43 ± 0.14
LN-ASK1	−2.20	0.45 ± 0.15
ASK1-LN	−2.12	0.46 ± 0.15
LC-ASK1	−2.29	0.45 ± 0.14
ASK1-LC	−2.16	0.46 ± 0.15

Quality parameters (C-score and estimated TM-value) of the 3D models for ASK1 fusion and Prdx1 fusion with mLumin are shown. Models with a C-score greater than −1.5 and an estimated TM-score greater than 0.5 presented a correct fold. Models with an estimated TM-score lower than 0.17 presented random similarity with the templates used for modeling. LN: large N-terminal half of mLumin. LC: small C-terminal half of mLumin.

**Table 2:** ASK1-LN interacting with Prdx1-LC gave the most stable and reliable docking, based on both the HADDOCK and Z-score.

ASK1 Fusion Proteins	Prdx1 Fusion Proteins	HADDOCK Score	Z-Score
ASK1-LN	LC-Prdx1	−360.0 ± 8.4	−1.8
ASK1-LN	Prdx1-LC	−362.1 ± 12.6	−2.2

The HADDOCK score and Z-score of each docking are shown. LN: large N-terminal half of mLumin. LC: small C-terminal half of mLumin.

**Table 3:** The rate constants for the interaction of Prdx variants with ASK1-TBD showed a peroxidatic cysteine-dependent fast reaction and the formation of a stable bond, suggesting a Prdx1-ASK1-TBD mixed disulfide.

Prdx Variants	k_on_ (M^−1^ s^−1^)	k_off_ (s^−1^)
Prdx1 WT	3.27 ± 0.87 × 10^4^	2.00 ± 0.76 × 10^−4^
Prdx1 C173A	0.72 ± 0.31 × 10^4^	2.28 ± 0.77 × 10^−3^
Prdx1 C52A	0	
Prdx2 WT	0	

## Data Availability

Data is contained within the article and Supplementary Material.

## Supplementary Materials

The following are available online at https://www.mdpi.com/article/10.3390/antiox10071060/s1, Table S1. List of primers used in the study. Figure S1. mLumin-fused Prdx1 WT, its mutants (C52A and C173A), Prdx2 WT, ASK1, and STAT3 are expressed at comparable levels between samples when co-transfected in HEK293 MSR and AnxA2 KO HEK293 MSR cells. Figure S2. GFP expression in HEK293 MSR cells. Figure S3. Recombinant Prdx1 WT, its mutants (C52A and C173A) and MBP-ASK1-TBD were successfully purified. Figure S4. Recombinantly expressed and purified Prdx1 WT and Prdx1 C173A display peroxidase activity, while the peroxidatic mutant Prdx1 C52A is not active. Figure S5. Prdx1 WT and Prdx1 C173A interact with ASK1-TBD in the presence of H2O2. Figure S6. The recombinant purified Prdx1 variants (C52A and C173A) maintain their secondary structure (compared to Prdx1 WT) in the reduced and oxidized form. Figure S7. GFP expression in AnxA2 KO HEK293 MSR cells.
