# Supplementary material for: Prdx1 Interacts with ASK1 upon Exposure to H2O2 and Independently of a Scaffolding Protein
Source: Antioxidants (Basel). 2021 Jun 30;10(7):1060. doi: 10.3390/antiox10071060 (PMC8300624; doi:10.3390/antiox10071060)
Supplement: Supplementary file 1 [file antioxidants-10-01060-s001.zip › antioxidants-1250810-supplementary.pdf]

Table S1. List of primers used in the study

**Purpose**	**Primer direction (forward: Fwd; reverse: Rev)**	**Sequence (5′ → 3′)**
Mutating the peroxidatic cysteine of Prdx1 (C52A) (codon-optimized for expression in mammalian cells)	Fwd	cttgacttcacctttgtggcccccacggagatcattgc
	Rev	gcaatgatctccgtgggggccacaaaggtgaagtcaag
Mutating the resolving cysteine of Prdx1 (C173A) (codon-optimized for expression in mammalian cells)	Fwd	gacaaacatggggaagtggccccagctggctggaaac
	Rev	gtttccagccagctggggccacttccccatgtttgtc
Mutating the peroxidatic cysteine of Prdx1 (C52A) (codon-optimized for expression in *E. coli*)	Fwd	gctggattttacctttgtggccccgaccgaaattattgcg
	Rev	cgcaataatttcggtcggggccacaaaggtaaaatccagc
Mutating the resolving cysteine of Prdx1 (C173A) (codon-optimized for expression in *E. coli*)	Fwd	gataaacatggcgaagtggccccggcgggctggaaacc
	Rev	ggtttccagcccgccggggccacttcgccatgtttatc
HiFi DNA assembly of the pcDNA3.1-ASK1-LN construct	pcDNA3.1-Fwd	actttcgaaacaaacagactggatcctatccttacgatgtgc
	pcDNA3.1-Rev	tcgtccgcctccgtgctcattctagagggcccgtttaaacg
	ASK1-Fwd	gtttaaacgggccctctagaatgagcacggaggcggacgag
	ASK1-Rev	acatcgtaaggataggatccagtctgtttgtttcgaaagtcaatg
HiFi DNA assembly of the pcDNA3.1-Prdx1-LC construct (same for Prdx1 WT, C52A, C173A)	pcDNA3.1-Fwd	aatatttctccaagcagaaggagcagaagctgatcagcgaag
	pcDNA3.1-Rev	ttagcatttcctgaagacatgcggccgctcgagtctagag
	Prdx1-Fwd	ctctagactcgagcggccgcatgtcttcaggaaatgctaaaattg
	Prdx1-Rev	tcgctgatcagcttctgctccttctgcttggagaaatattctttgctcttt

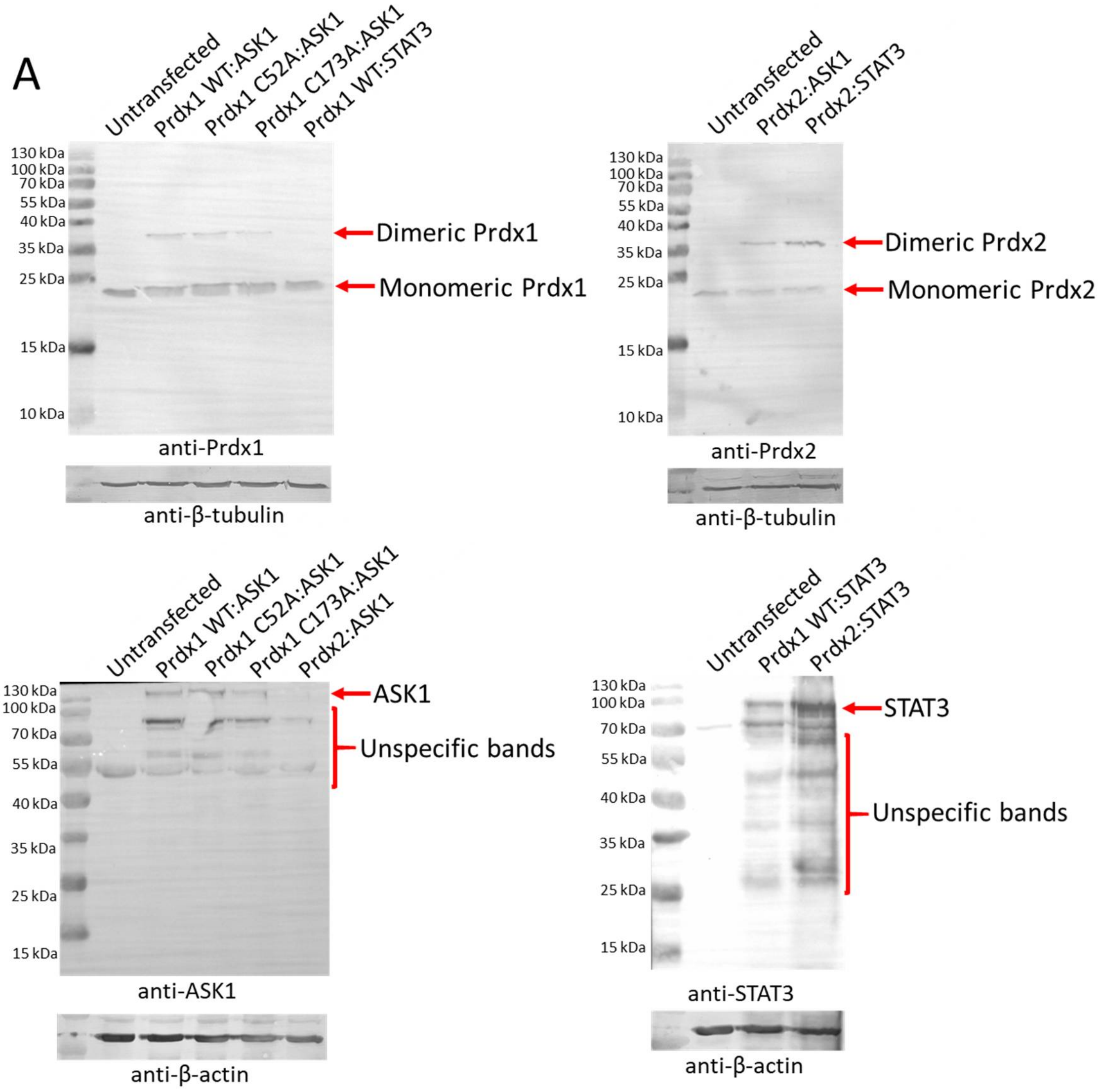
A
Untransfected
Prdx1 WT:ASK1
Prdx1 C52A:ASK1
Prdx1 C173A:ASK1
Prdx1 WT:STAT3
130 kDa
100 kDa
70 kDa
55 kDa
40 kDa
35 kDa
25 kDa
15 kDa
10 kDa
Dimeric Prdx1
Monomeric Prdx1
anti-Prdx1
anti-β-tubulin
Untransfected
Prdx2:ASK1
Prdx2:STAT3
Dimeric Prdx2
Monomeric Prdx2
anti-Prdx2
anti-β-tubulin
Untransfected
Prdx1 WT:ASK1
Prdx1 C52A:ASK1
Prdx1 C173A:ASK1
Prdx2:ASK1
ASK1
Unspecific bands
anti-ASK1
anti-β-actin
Untransfected
Prdx1 WT:STAT3
Prdx2:STAT3
STAT3
Unspecific bands
anti-STAT3
anti-β-actin

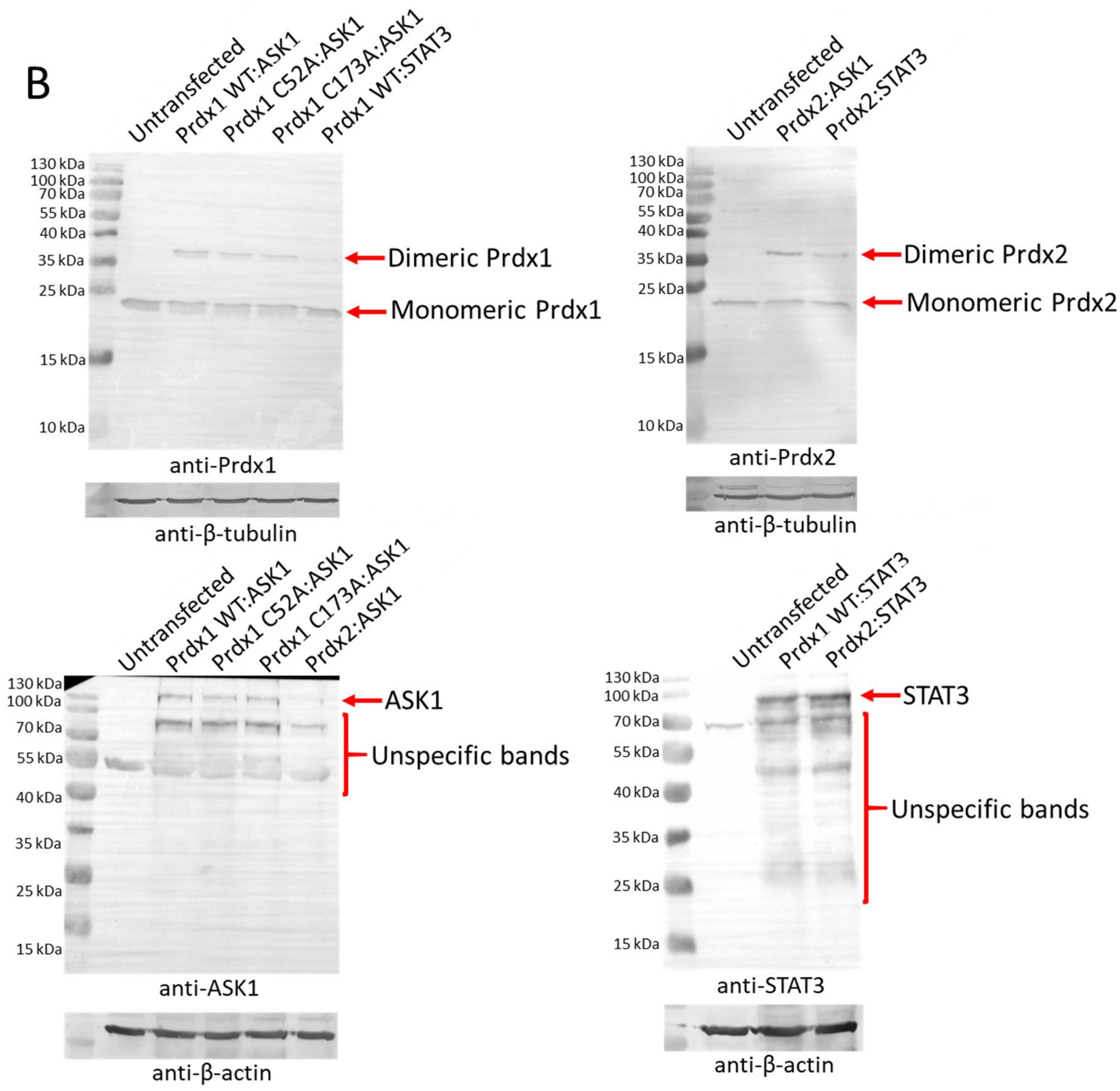

Figure S1. mLumin-fused Prdx1 WT, its mutants (C52A and C173A), Prdx2 WT, ASK1, and STAT3 are expressed at comparable levels between samples when co-transfected in HEK293 MSR and AnxA2 KO HEK293 MSR cells. Antibodies used in the western blot (reducing): rabbit anti-Prdx1, rabbit anti-Prdx2, mouse anti-ASK1, mouse anti-STAT3, rabbit β-tubulin and mouse β-actin. (**A**) Protein expression in HEK293 MSR cells. (**B**) Protein expression in AnxA2 KO HEK293 MSR cells. β-tubulin and β-actin were used as house-keeping proteins.

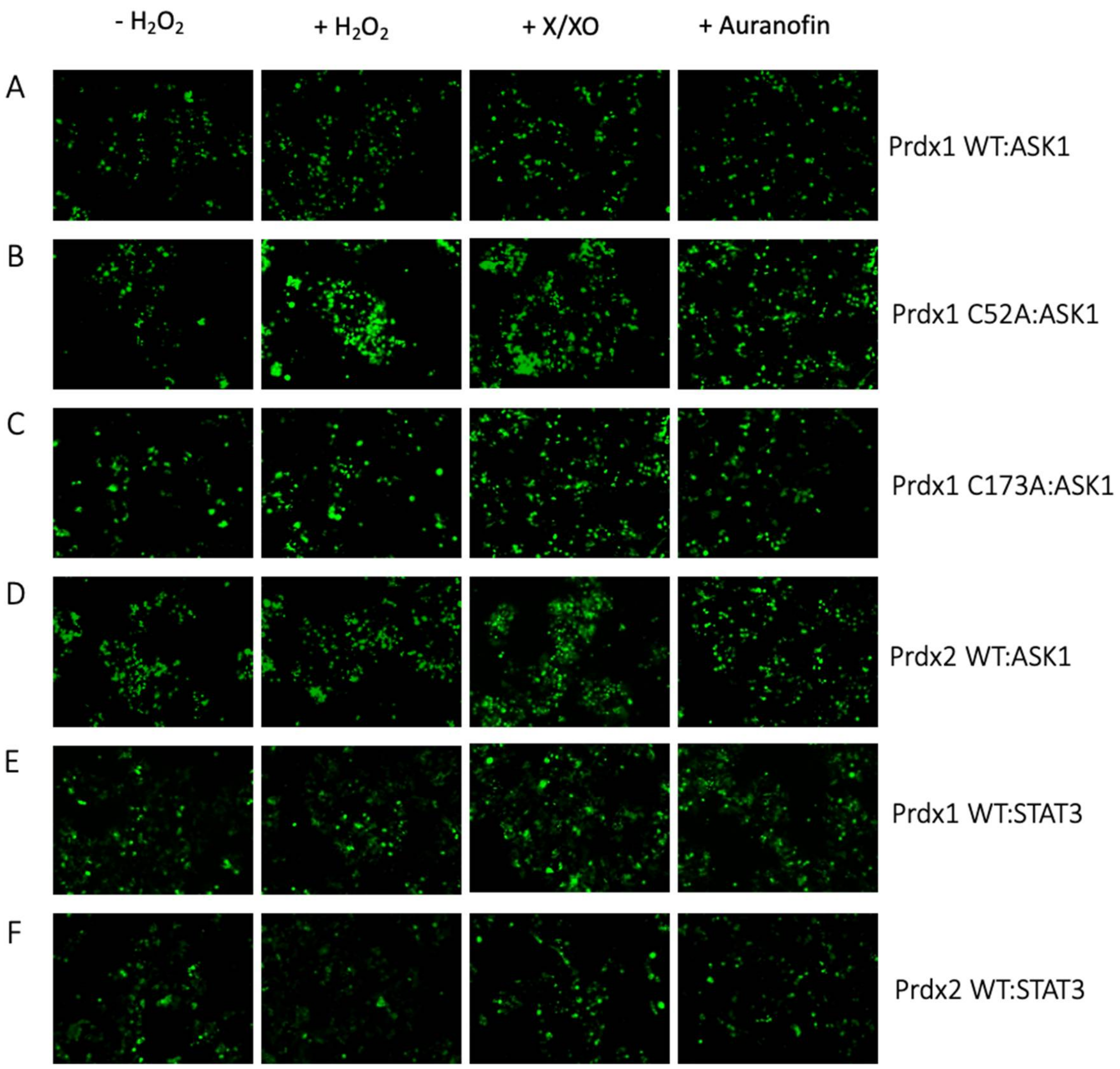

Figure S2. GFP expression in HEK293 MSR cells. The green dots are the fluorescence signal emitted by GFP, which was used for normallization of the mLumin signal in Figure 3. The cells have been transfected with constructs to monitor the (**A**) Prdx1 WT:ASK1 (**B**) Prdx1 C52A:ASK1 (**C**) Prdx1 C173A:ASK1 (**D**) Prdx2 WT:ASK1. (**E**) Prdx1 WT:STAT3 (**F**) Prdx2 WT:STAT3 interaction. The treatments from left to right are: no treatment, 100 μM H_2O_2 for 30 min, X/XO (8 μM X and 1 mU/mL XO) for 18 h and 0.8 μM auranofin for 18 h.

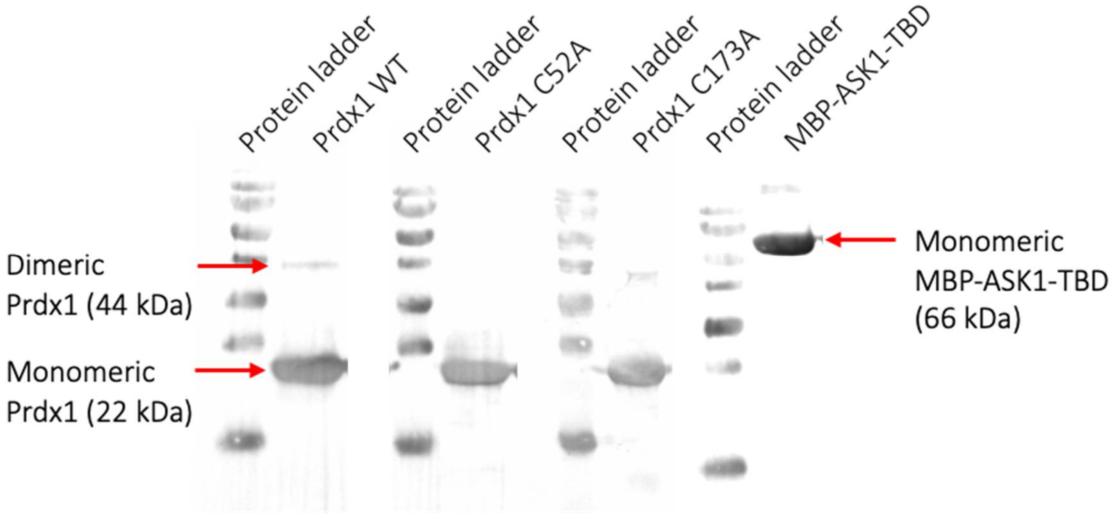

Figure S3. Recombinant Prdx1 WT, its mutants (C52A and C173A) and MBP-ASK1-TBD were successfully purified. Antibodies used in the western blot (non-reducing): rabbit anti-Prdx1 and mouse anti-His for His-MBP-ASK1-TBD.

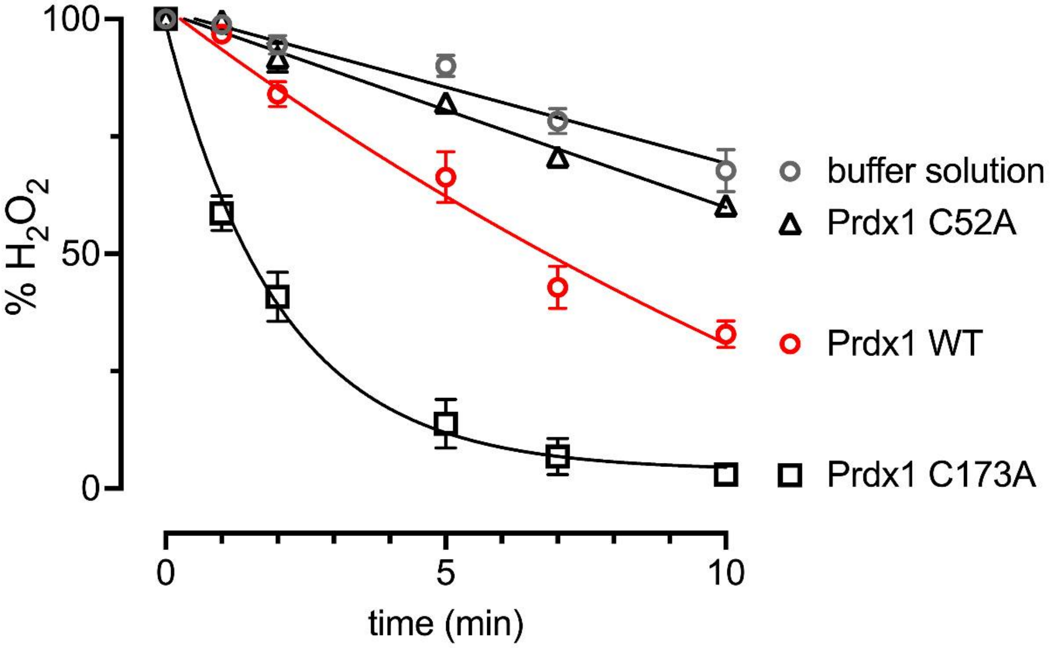

Figure S4. Recombinantly expressed and purified Prdx1 WT and the resolving Cys mutant Prdx1 C173A display peroxidase activity, while the peroxidatic mutant Prdx1 C52A is not active. The H_2O_2 concentration was determined using a FOX assay by taking samples at different time points after incubating 2 μM of the Prdx1 variants with 200 μM H_2O_2. 1 μM DTT was used as an electron donor. Data were fitted with a single exponential decay. Prdx1 WT is more sensitive to overoxidation than its resolving cysteine mutant (C173A). The results are representative of at least three independent experiments. The error bars represent the standard deviation.

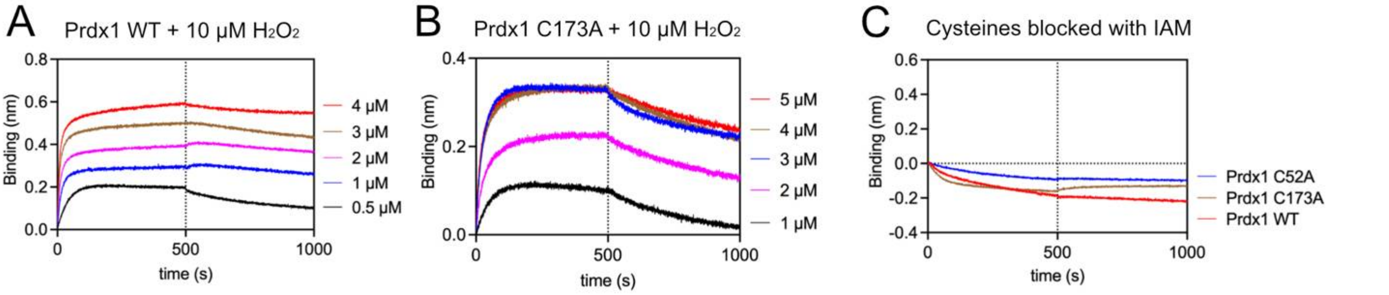

Figure S5 Prdx1 WT and Prdx1 C173A interact with ASK1-TBD in the presence of H_2O_2. The interaction of increasing concentrations of (**A**) Prdx1 WT and (**B**) Prdx1 C173A with ASK1-TBD in the presence of 10 μM H_2O_2. Based on these curves the k_{on} and k_{off} reaction rates have been determined. (**C**) The Prdx1:ASK1 interaction is completely abolished after IAM blocking of the cysteines of the Prdx1 variants and ASK1-TBD. MBP was used as a reference. In all cases, the displayed results are obtained after subtracting the binding to MBP. The Y-axis indicates the wavelength shift (in nm) of the Octet[Red] 96 instrument. The vertical dashed line separates the association from dissociation phase.

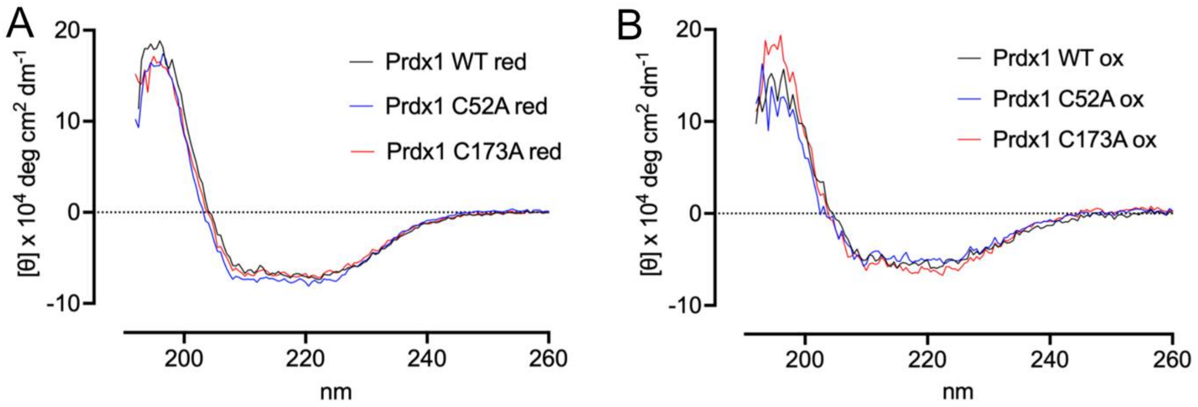

Figure S6. The recombinant purified Prdx1 variants (C52A and C173A) maintain their secondary structure (compared to Prdx1 WT) in the reduced and oxidized form. (**A**) Prdx1 WT and its mutant circular dichroism spectra in the reduced state. (**B**) Prdx1 WT and its variant circular dichroism spectra upon oxidation by a 10-fold molar ratio of H_2O_2 to Prdx1. The Y-axis represents the molar ellipticity of the indicated proteins.

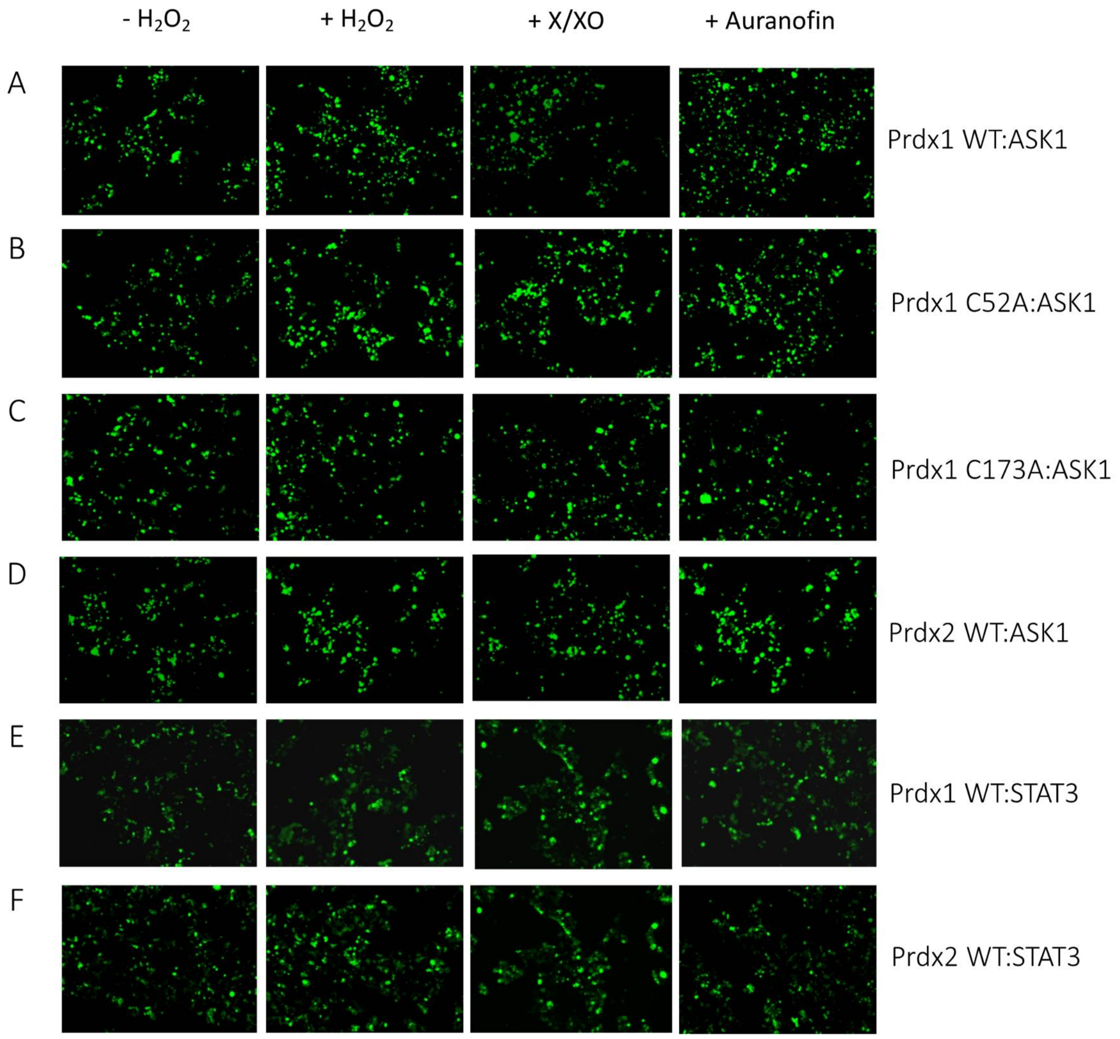

Figure S7. GFP expression in AnxA2 KO HEK293 MSR cells. The green dots are the fluorescence signal emitted by GFP, which was used for normallization of the mLumin signal in Figure 5. The cells have been transfected with constructs to monitor the (**A**) in Prdx1 WT:ASK1 (**B**) in Prdx1 C52A:ASK1 (**C**) in Prdx1 C173A:ASK1 (**D**) in Prdx2 WT:ASK1 (**E**) in Prdx1 WT:STAT3 (**F**) in Prdx2 WT:STAT3 interaction. The treatments from left to right are: no treatment, 100 μM H_2O_2 for 30 min, X/XO (8 μM X and 1 mU/mL XO) for 18 h and 0.8 μM auranofin for 18 h.
